# Observations on *Pluteaceae* in Vietnam: Four New Species and New Records of *Pluteus*

**DOI:** 10.3390/jof9050584

**Published:** 2023-05-18

**Authors:** Ekaterina Malysheva, Eugene Popov, Olga Morozova, Vasily Dudka, Thi Ha Giang Pham, Vera Malysheva

**Affiliations:** 1 Komarov Botanical Institute of the Russian Academy of Sciences, Prof. Popov Str. 2, 197022 St. Petersburg, Russia; epopov@binran.ru (E.P.); omorozova@binran.ru (O.M.); vdudka@binran.ru (V.D.); v_malysheva@binran.ru (V.M.); 2 Joint Vietnam-Russia Tropical Science and Technology Research Centre, 63 Nguyen Van Huyen Str., Cau Giay, Hanoi 122000, Vietnam; giangvietnga@gmail.com

**Keywords:** Agaricales, Basidiomycota, molecular phylogeny, new species, nrITS, *tef1*, taxonomy

## Abstract

Eighteen specimens of *Pluteus* collected from the tropical forests of Vietnam were studied using morphological and molecular approaches. *Pluteus podospilloides*, *P. semibulbosus*, *P. chrysaegis* and *P. septocystidiatus* are registered as additional or new records for Vietnam. Four species (*P. conformis*, *P. lucidus*, *P. subroseus*, and *P. ornatus*) are proposed as new to science, and several other collections (*Pluteus* sp. 1, *P.* aff. *septocystidiatus*, *P.* aff. *pauperculus* and *P.* cf. *velutinus*) are given an inconclusive taxonomic status for now. The taxonomic positions of all specimens were confirmed using DNA data (nrITS and *tef1*). Descriptions of the macro- and microscopic features of the studied collections with a discussion of similar taxa are given.

## 1. Introduction

This paper is part of a series dealing with the members of the *Pluteaceae* family in Vietnam [1,2,3].

The genus *Pluteus* Fr. is very species-rich and widespread from the Arctic to tropical areas [4,5,6,7,8]. It is characterized by basidiocarps without volva, free lamellae, pink or pinkish brown spore print, smooth and inamyloid basidiospores, and inverse hymenophoral trama. According to recent morphological and molecular studies [6,7], three sections within the genus *Pluteus* were confirmed: sect. *Pluteus* characterized by metuloid pleurocystidia and a pileipellis organized as a cutis; sect. *Celluloderma* which included members with the non-metuloid (or absent) pleurocystidia and a pileipellis as a hymeniderm or epithelium; and sect. *Hispidoderma* which included species with the non-metuloid pleurocystidia and hymeniderm or trichoderm pileipellis. More than 500 species of the genus have been described worldwide [9]. However, the species diversity of this genus has not yet been comprehensively studied in Vietnam. Prior to our study, only four species of *Pluteus*—*P. cervinus* (Schaeffer) Kummer, *P. minutus* Pat., *P. plautus* (Weinm.) Gillet and *P. semibulbosus* (Lasch) Quél.—were previously reported so far from the country [10,11,12]. Our recent studies have shown the presence of ten previously undescribed *Pluteus* species in the mycobiota of Vietnam [2]. With the addition of the species described below, the number of *Pluteus* species on the studied territory increased to twenty-three.

In this article four new species are described below, detailed descriptions and illustrations of new taxa and new records are given, and the phylogenetic relationships of the identified species and related taxa from the genus Pluteus are analyzed on the basis of molecular data.

## 2. Materials and Methods

### 2.1. Morphological Examination

Macroscopic descriptions were based on fresh basidiocarps from the original collections and photos were taken at the site. The RAL color standard (K7 color fan, edition 2019; https://www.ral-farben.de/en/ral-k7-color-fan, accessed on 1 March 2023) was used for basidiocarps description. The collections were examined and drawn using standard microscopic techniques [13]. Microscopic observations were conducted via dried material mounted in 5% KOH and Congo Red using an Axio Imager.A1 light microscope (CarlZeiss, Oberkochen, Germany) equipped with differential interference contrast optics (DIC). For statistical evaluation of basidiospore dimensions, at least 30 basidiospores were measured from each basidiocarp; [60/2/1] indicates measurements based on 60 basidiospores from 2 basidiocarps in 1 collection. Basidiospore dimensions are given in the following form: (a)b–c(d), with b–c containing at least 90% of all values, and the extremes (a, d) enclosed in parentheses; Lav and Wav are the mean value of length and width of the total basidiospores measured, respectively. Q indicates the basidiospore length/width ratio, and Qav represents the mean length/width quotient of the total basidiospores measured. All morphological terms were used following Vellinga [14].

The collected specimens were deposited in the Mycological Herbarium of the Komarov Botanical Institute, Saint Petersburg (LE F) and in the Herbarium of the Joint Vietnam-Russia Tropical Science and Technology Research Centre, Hanoi (VRTC).

### 2.2. Molecular Techniques

For DNA extraction, small fragments of dried basidiocarps were used. The procedure of DNA extraction completely corresponded to the manufacturer’s protocol of the Phytosorb Kit (ZAO Syntol, Moscow, Russia). The following primers were used for amplification and sequencing: ITS1F-ITS4B [15,16] for the ITS1-5.8S-ITS2 fragment; and EF1-983F and EF1-1567R for approximately 500 bp of *tef1* [17]. PCR products were purified by applying the GeneJET Gel Extraction Kit (Thermo Scientific, Thermo Fisher Scientific Inc., Waltham, MA, USA). Sequencing was performed with an ABI model 3500 Genetic Analyzer (Applied Biosystems, Carlsbad, CA, USA). Raw data were edited and assembled in MEGA X [18].

All microscopic and molecular studies of specimens were carried out at the Center for collective use of scientific equipment “Cellular and molecular technology of studying plants and fungi” (Komarov Botanical Institute, Russian Academy of Sciences, St. Petersburg, Russia).

### 2.3. Phylogenetic Analyses

For this study, 17 new nrITS sequences and 13 *tef1* sequences were generated. In addition, 197 nrITS sequences and 52 *tef1* sequences, including outgroups, were retrieved from the GenBank database (www.ncbi.nlm.nih.gov/genbank/, accessed on 25 March 2023), using the BLAST application and taxonomic considerations. The sequences from the GenBank database and previous studies (with ≥60% query coverage and ≥85–100% sequence similarity) were selected and listed in Table S1. The taxonomic identities of these sequences are given in phylogenetic trees as they appear in GenBank. The sequences were aligned with the Muscle tool incorporated into the MEGA X program.

Phylogenetic reconstructions were performed for three data sets (sect. *Pluteus*, sect. *Celluloderma* and sect. *Hispidoderma*) with Maximum Likelihood (ML) and Bayesian Inference (BI) analyses. Before the analyses, the best-fit substitution models were estimated separately for three data sets based on the Akaike Information Criterion (AIC) using the FindModel web server (http://www.hiv.lanl.gov/content/sequence/findmodel/findmodel.html, accessed on 25 March 2023).

For the sect. *Pluteus*, we performed a phylogenetic analysis based on two genetic markers (nrITS+tef1). The GTR+G model was chosen for nrITS dataset and K80+G for *tef1*. Hence, we used a partitioned evolution model for concatenated nrITS+*tef1* dataset in ML and Bayesian analyses to reconstruct a robust phylogenetic hypothesis. Maximum likelihood analyses were run on RAxML servers, v.1.0.0 (https://raxml-ng.vital-it.ch/#/, accessed on 25 March 2023) with 1000 bootstrap replicates. BI analyses were performed with MrBayes 3.2.5 software [19], for two independent runs, each with 7 million generations under described models and four chains with sampling every 100 generations. To check for convergence of MCMC analyses and to get estimates of the posterior distribution of parameter values, Tracer v1.7.1 was used [20]. We accepted the result where the ESS (Effective Sample Size) was above 200 and the PSRF (Potential Scale Reduction Factor) was close to 1. Branches with bootstrap support (BS) and posterior probabilities (PP) values greater than or equal to 70% and 0.95, respectively, were considered to be significantly supported.

For sect. *Celluloderma* and sect. *Hispidoderma*, phylogenetic reconstructions were based only on nrITS, because of the lack of a satisfactory data set in the GenBank for the second marker *tef1*. The same GTR+G model was chosen for both nrITS alignments. The ML and BI analyses were conducted similarly as the pattern for section *Pluteus*.

Pairwise distances between the nr ITS sequences were calculated using p-distance methods and the maximum composite likelihood model in the MEGA X program.

Newly generated sequences were deposited in GenBank with corresponding accession numbers (Table S1).

## 3. Results

### 3.1. Phylogeny

The combined data set of nrITS+*tef1* sequences for members of sect. *Pluteus* contained 1244 characters, including gaps (nrITS: 1–660 and *tef1*: 661–1244). The nrITS data set for sect. *Celluloderma* contained total 709 characters and for sect. *Hispidoderma* there were 787 characters, including gaps. The overall topologies of the ML and BI trees were nearly identical for all data sets. Therefore, we present a single phylogram for each section with bootstrap values ≥ 70% and a posteriori probability ≥ 0.90.

**Figure 1:**
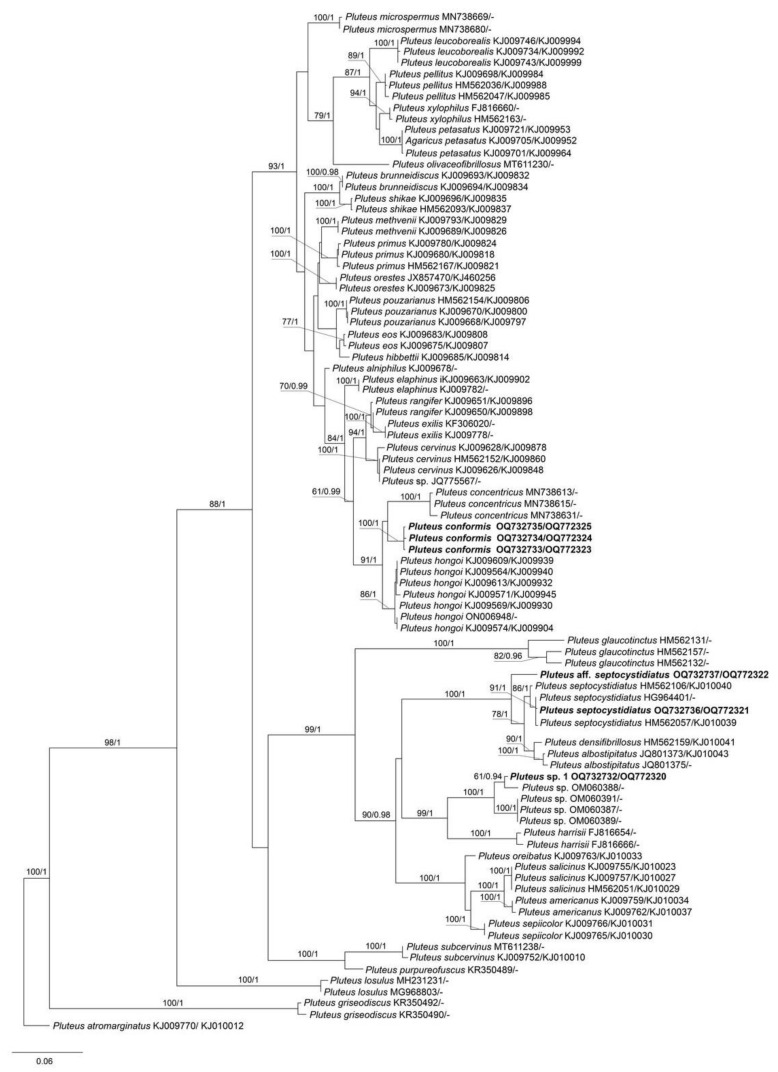
Best tree from the ML analysis for *Pluteus* sect. *Pluteus* species of the nrITS+*tef1* data set, with *Pluteus atromarginatus* as outgroup. Bootstrap support and Posterior probability values (BS/PP) are given above the branches. All sequences are labeled with taxon name and GenBank accession numbers nrITS/*tef1*. The sequences generated in the present study are in bold.

The output ML tree of the phylogenetic analyses for sect. *Pluteus* comprised 83 specimens of *Pluteus* and 1 specimen of the outgroup (*P. atromarginatus* (Konrad) Kühner, LE F-289425). Most of *Pluteus* species included in the analyses form strongly supported clades, which is in agreement with the earlier studies [6,7,8,21,22]. Our phylogenetic results (Figure 1) confirmed that three Vietnamese collections (LE F-313663, 313664 and 313665) formed a highly supported (BS = 100% and PP = 1.0) monophyletic clade, which is a sister lineage to the *P. concentricus* clade, and clearly separated from *P. hongoi*. We describe them below as a new species, *P. conformis*. Two collections (LE F-313667, and 313668) belongs to *P. septocystidiatus*/*P. albostipitatus* clade, and this placement is strongly supported in the tree (Figure 1). Another studied Vietnamese collection (LE F-313670) was unexpectedly phylogenetically close to several collections from Vanuatu, which represent a species new to science (under description and publication by Jonathan Andrew del Rosario).

**Figure 2:**
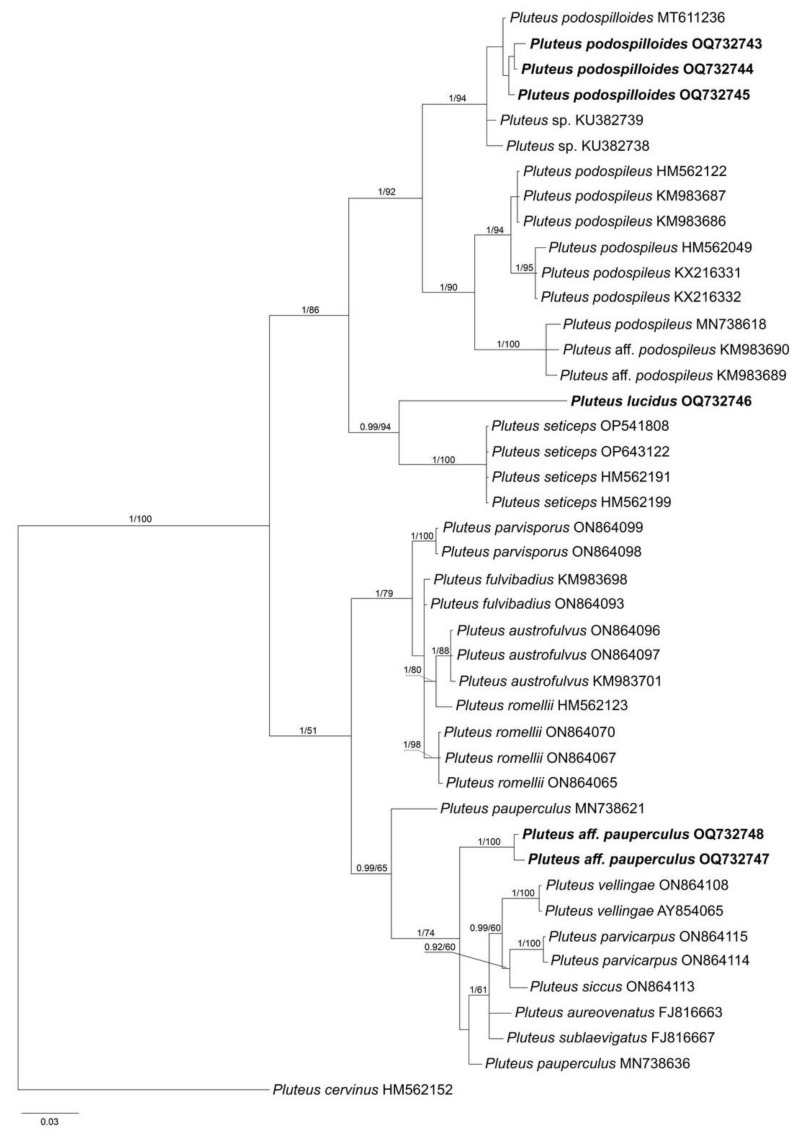
Phylogenetic tree inferred from a Bayesian analysis for *Pluteus* sect. *Celluloderma* species of the nrITS data set, with *Pluteus cervinus* as outgroup. Posterior probability and Bootstrap support values (PP/BS) are given above the branches. All sequences are labeled with taxon name and GenBank accession number. The sequences generated in the present study are in bold.

The output BI tree of the phylogenetic analyses for sect. *Celluloderma* comprised 42 specimens (data set was restricted by all available sequences phylogenetically close to the studied collections) and 1 specimen of the outgroup (*P. cervinus*, REG 13641). As Figure 2 suggests, the studied Vietnamese collections fall in three lineages: three specimens (LE F-347430, 347431, and 313666), representing additional finds of *P. podospilloides* E.F. Malysheva et O.V. Morozova and are nested in the same clade as the holotype of the species; newly described species, *P. lucidus,* occupies a sister position to *P. seticeps* (G.F. Atk.) Singer; and *P.* aff. *pauperculus* is close to *P. pauperculus* E. Horak and is clustered in a well-supported group that harbors species from the romellii clade [23].

**Figure 3:**
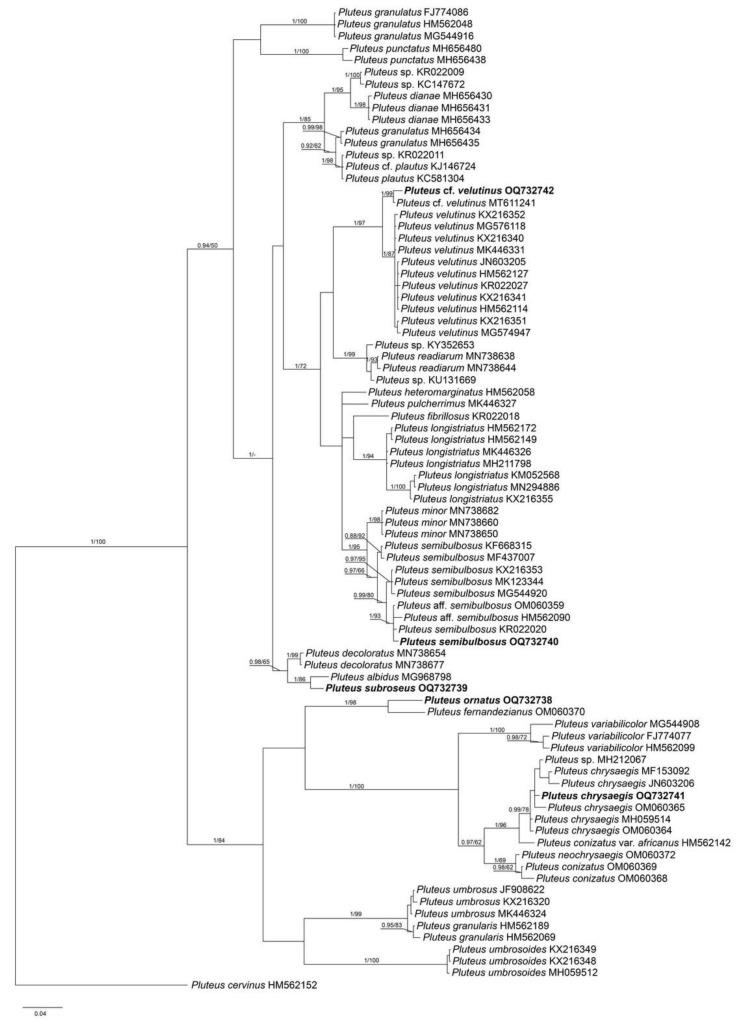
Phylogenetic tree inferred from a Bayesian analysis for *Pluteus* sect. *Hispidoderma* species of the nrITS data set, with *Pluteus cervinus* as outgroup. Posterior probability and Bootstrap support values (PP/BS) are given above the branches. All sequences are labeled with taxon name and GenBank accession number. The sequences generated in the present study are in bold.

The output BI tree of the phylogenetic analyses for sect. *Hispidoderma* comprised 83 specimens, including the outgroup (*P. cervinus*, REG 13641). The nrITS sequences of *P.* cf. *velutinus* cluster together with *P. velutinus* C.K. Pradeep, Justo et K.B. Vrinda on a highly supported (PP = 1.00, BS = 97%) clade in all analyses. Two Vietnamese collections (LE F-313653 and LE F-347436), representing known taxa *P. semibulbosus* and *P. chrysaegis* (Berk. et Broome) Petch, are nested in the corresponding monophyletic clades with high support (Figure 3). The collection of newly described species, *P. subroseus,* is grouped together with a single available sequence of *P. albidus* Beeli in one clade with high support (PP = 1.00, BS = 86%). However, nrITS sequences of these specimens contain a large number of unique characters (14 differences of 632 b. p., genetic distance is greater than 2%) which could be a reason of considering them as separate taxa. Furthermore, significant morphological differences were found between these two specimens. Another new species, *P. ornatus*, was recovered as a sister lineage of *P. fernandezianus* Singer and differ from the latter in 36 b. p. of 656 total b. p. (2.7% difference). Because this genetic distance is seemingly congruous to the morphological differences, we regard it as a sufficient species-specific difference and introduce a new species, *P. ornatus*, based on studied Vietnamese collections.

### 3.2. Taxonomy

#### 3.2.1. Sect. *Pluteus*

**Figure 4:**
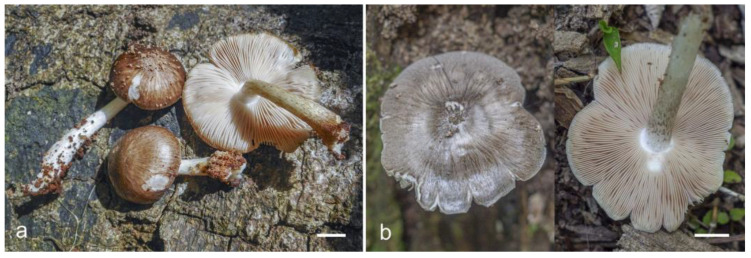
Basidiocarps of *Pluteus conformis* in situ: (**a**) holotype, LE F-313664; (**b**) LE F-313663. Scale bars 1 cm.

**Figure 5:**
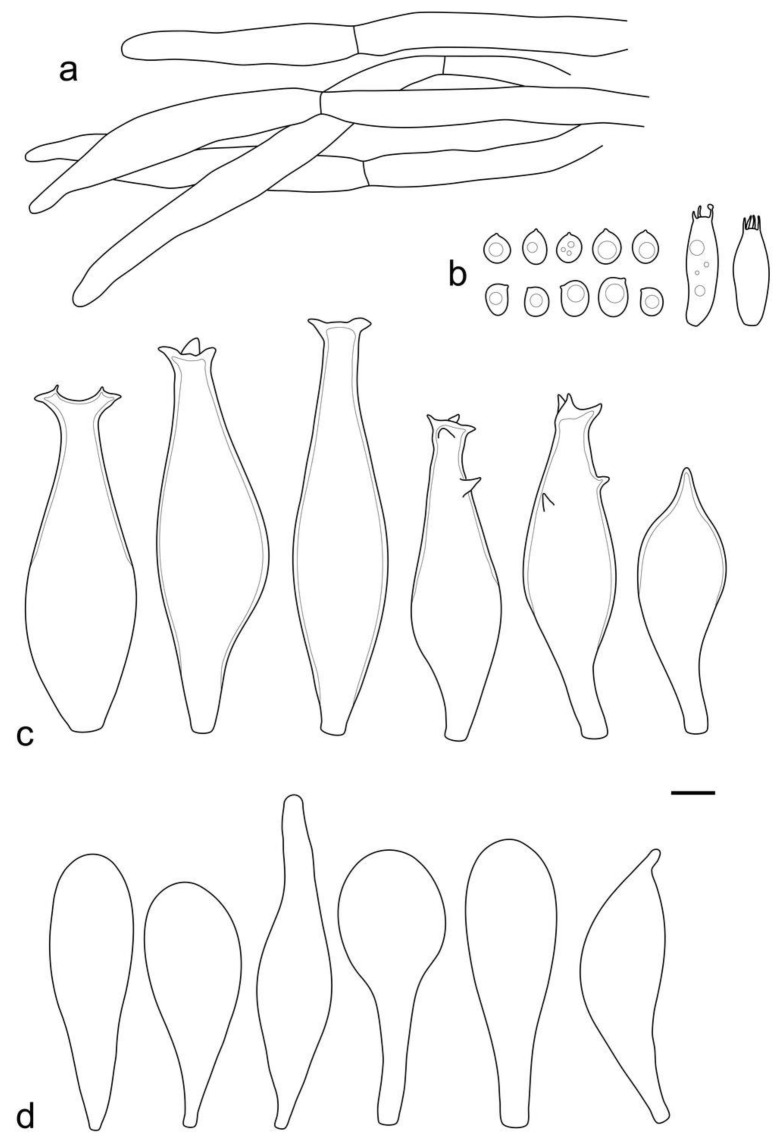
*Pluteus conformis* holotype (LE F-313664) microcharacters: (**a**) pileipellis elements; (**b**) basidia and spores; (**c**) pleurocystidia; (**d**) cheilocystidia. Scale bar 10 μm.

***Pluteus conformis*** E.F. Malysheva, sp. nov. (Figure 4 and Figure 5).

MB 848250.

Etymology: the specific epithet “conformis” (similar) emphasizes the similarity with *P. hongoi* and the rest of the species from the *P. cervinus* complex, for which morphological diversification of species is very difficult.

Diagnosis: differs from *P. hongoi* as it is characterized by the distinct longitudinal fibrils on stipe and smaller basidiospores.

Holotype: Vietnam, Lao Cai Province, Bat Xat District, Y Ty commune, Nui Co San range, Bat Xat Nature Reserve, 22°37′09.973″ N, 103°37′24.607″ E, h = 1880 m.a.s.l., disturbed montane tropical mixed forest, fallen trunk, on rotten wood, 21 April 2021, col. E.S. Popov (LE F-313664).

Pileus 25–50 mm in diam., initially hemispherical or campanulate, then plano-convex or applanate with or without broad low umbo; not hygrophanous, not striate; pale brown (RAL 8025), beige grey (RAL 7006), or clay brown (RAL 8003) to copper brown (RAL 8004), with concolorous or slightly darker center; surface dry, radially fibrillose, squamulose at the center. Lamellae free, crowded, with lamellulae, slightly ventricose, at first whitish then pale pinkish, with concolorous even edges. Stipe 30–60 × 3–7 mm, broadened towards base but without distinct bulb, solid, white, with sparse, longitudinal brown or grey-brown fibrils all over the surface. Smell raphanoid, taste not recorded.

Basidiospores [90/3/3] 5.4–7.5 × 4.4–5.7 µm (Lav = 6.4, Wav = 4.9, Q = 1.2–1.5; Qav = 1.3), broadly ellipsoid or oblong, sometimes cylindrical or ovoid, thick-walled. Basidia 22–25 × 7.7–8.5 µm, 4-spored. Pleurocystidia metuloid, 71–91 × 17.5–26.0 µm, numerous, narrowly to broadly fusiform, with 3–5 apical, predominantly bifid hooks, sometimes with lateral hooks, hyaline, thick-walled (with wall up to 2.5–3 µm). Intermediate cystidia similar to the pleurocystidia but smaller, broadly fusiform, often with single apical hook or without distinct apical hooks. Cheilocystidia (36–)46–70 × 16–25 µm, abundant, forming a sterile layer at the edge of lamellae, broadly clavate or spheropedunculate, hyaline, slightly thick-walled. Pileipellis a cutis, with cylindrical or narrowly fusiform, somewhat ascending terminal elements, 95–110 × 8–11 µm, with brown intracellular pigment, thin- or slightly thick-walled. Stipitipellis a cutis of cylindrical, slightly thick-walled, 7–15 µm wide hyphae, hyaline or with brown intracellular pigment, some incrusted. Caulocystidia absent. Clamp connections absent in all tissues.

Habitat: solitary or in small groups, on rotten wood.

Additional collections examined: Vietnam, Lao Cai Province, Bat Xat District, Den Sang commune, Bat Xat Nature Reserve, 22°37′26.44″ N, 103°38′07.645″ E, h = 1794 m.a.s.l., disturbed montane tropical broad-leaved forest, on strongly rotten stump, 20 April 2021, col. Đỗ Tất Thịnh (LE F-313665, duplicate in VRTC—ESP-005280); Lao Cai Province, Bat Xat District, Den Sang commune, Bat Xat Nature Reserve, 22°37′29.064″ N, 103°37′42.456″ E, h = 1835 m.a.s.l., disturbed montane tropical forest, on rotten wood, 23 April 2021, col. V.A. Dudka (LE F-313663).

Notes: *Pluteus conformis* is introduced here based on three specimens, all collected from the northern Province of Vietnam in montane tropical broad-leaved forest. It is mostly similar to *P. hongoi* Singer, but the presence of distinct longitudinal fibrils on stipe and smaller basidiospores makes it different from the latter (vs. 5.5–9.0 × 4.5–7.0 μm in *P. hongoi* [8]). Molecular data (Figure 1) supports the separation of both taxa.

*Pluteus concentricus* E. Horak comes very close to *P. conformis* in the molecular analyses. It differs mainly due to the pigmented lamellae edges, the presence of the characteristic concentric bulges on pileus, smaller basidiospores (less than 6.5 μm), and its distribution range, which is restricted to New Zealand [24].

**Figure 6:**
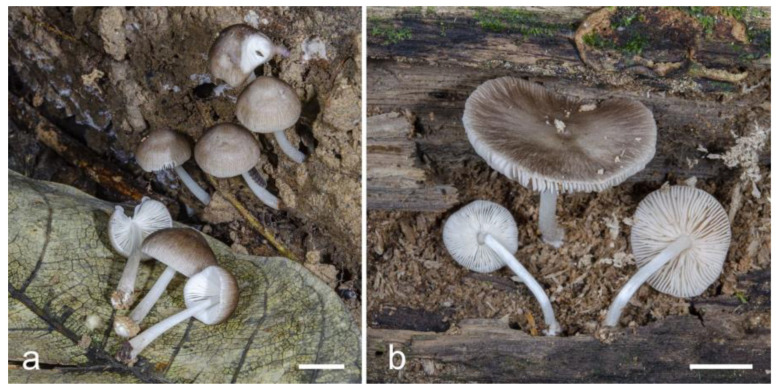
Basidiocarps in situ: (**a**) *Pluteus septocystidiatus* (LE F-313668); (**b**) *Pluteus* aff. *septocystidiatus* (LE F-313667). Scale bars 1 cm.

**Figure 7:**
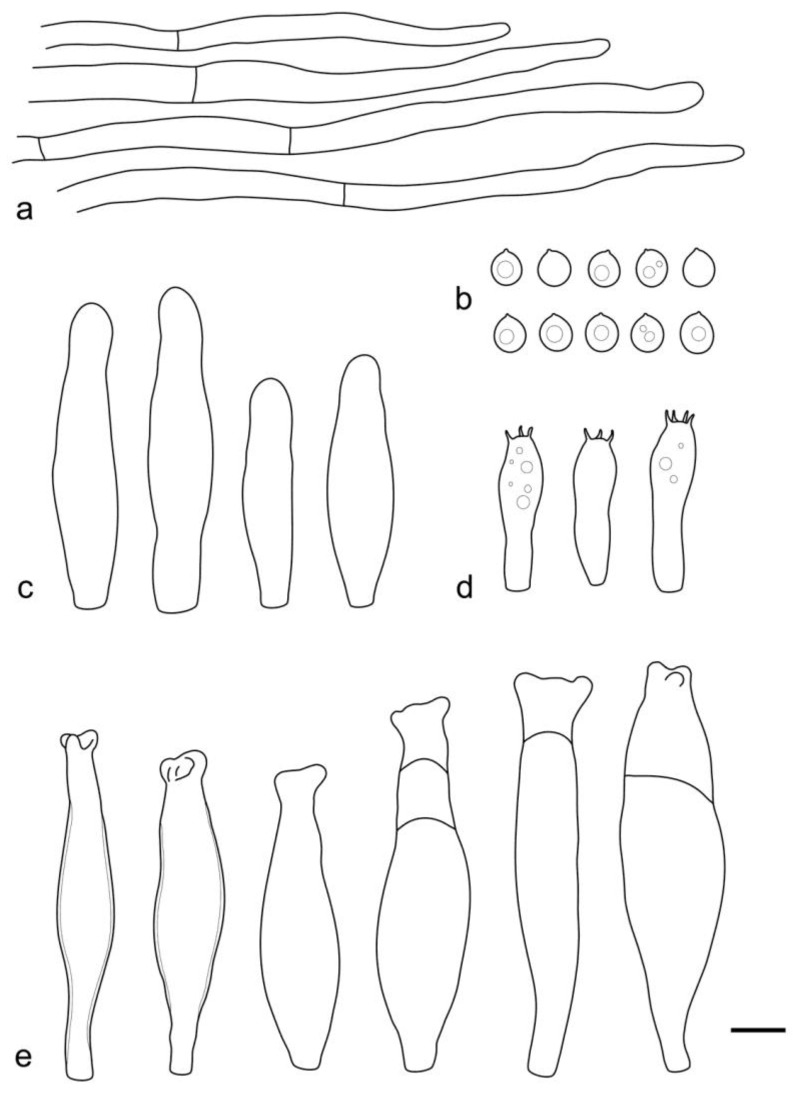
*Pluteus septocystidiatus* (LE F-313668) microcharacters: (**a**) pileipellis elements; (**b**) spores; (**c**) cheilocystidia; (**d**) basidia; (**e**) pleurocystidia. Scale bar 10 μm.

***Pluteus septocystidiatus*** Ševčíková, Antonín et Borov., Sydowia 66(2): 230 (2014) (Figure 6 and Figure 7).

Pileus 10–20 mm in diam., when young hemispherical or campanulate, expanding to convex with broad low umbo; slightly hygrophanous, translucently striate to the half of the radius; pale brown (RAL 8025) or beige brown (RAL 8024); surface radially fibrillose. Lamellae free, moderately distant, ventricose, pink, with concolorous even edges. Stipe 30–50 × 2.5–4.0 mm, almost cylindrical or slightly broadened towards base but without bulb, white or light ivory (RAL 1015), glabrous or slightly pruinose at base. Smell indistinct, taste not recorded.

Basidiospores [60/2/1] 5.3–7.0 × 4.8–5.7 µm (Lav = 6.0, Wav = 5.2, Q = 1.1–1.3; Qav = 1.2), predominantly globose or subglobose, some broadly ellipsoid, thick-walled. Basidia 27–33 × 7.5–8.5 µm, 4-spored, but sometimes 1-spored, broadly clavate. Pleurocystidia 59–72 × 10–17 µm, rather numerous, narrowly fusiform or fusiform, with apical obtuse undeveloped hooks, predominantly septate, but non-septate cystidia also present but rare, hyaline, thin- or slightly thick-walled. Cheilocystidia 42–70 × 10–14 µm, scarce, narrowly clavate or cylindrical with obtuse apex, hyaline, thin-walled. Pileipellis a cutis, made up of cylindrical hyphae 7–12 µm wide, with brown intracellular, sometimes incrusted, pigment, thin- or slightly thick-walled. Stipitipellis a cutis of cylindrical, hyaline, slightly thick-walled, 5–10 µm wide hyphae. Caulocystidia not seen. Clamp connections absent.

Habitat: in small groups, on rotten wood.

Collections examined: Vietnam, Nghe An Province, Con Cuong District, Chau Khe commune, 2.5 km WNW of Khe Choang ranger station, Pu Mat National Park, 18°57′21.564″ N, 104°41′06.431″ E, h = 204 m.a.s.l., disturbed lowland tropical broad-leaved monsoon forest, on rotten wood, 20 April 2018, col. E.S. Popov (LE F-313668, duplicate in VRTC—ESP-003808).

Notes: *Pluteus septocystidiatus*, originally described based on collections from the Republic of Korea and the USA, is characterized by high variability of both macro- and microscopic morphological characteristics [25]. Nevertheless, the main distinguishing features of the species are recognized to be thick-walled and septate pleurocystidia, sometimes septate cheilocystidia, and strongly striate and concave pileus. The Vietnamese specimen that we studied, being phylogenetically identical to the holotype of *P. septocystidiatus* and included in the same clade with high support (Figure 1), is morphologically different from the previously studied collections of this species. It is characterized by the presence of campanulate and convex pileus and slightly smaller basidiospores (vs. (5.5–)6.0–8(–9) × 5.5–7.5(–8) µm). Based on the above features, our specimen is also difficult to distinguish from the morphologically close species, *P. albostipitatus* (Dennis) Singer, which, however, is phylogenetically distinct and occupies a sister position on the phylogenetic tree (Figure 1).

Thus, our finding extends the geographic distribution of the species and the range of variation in its morphological characteristics.

**Figure 8:**
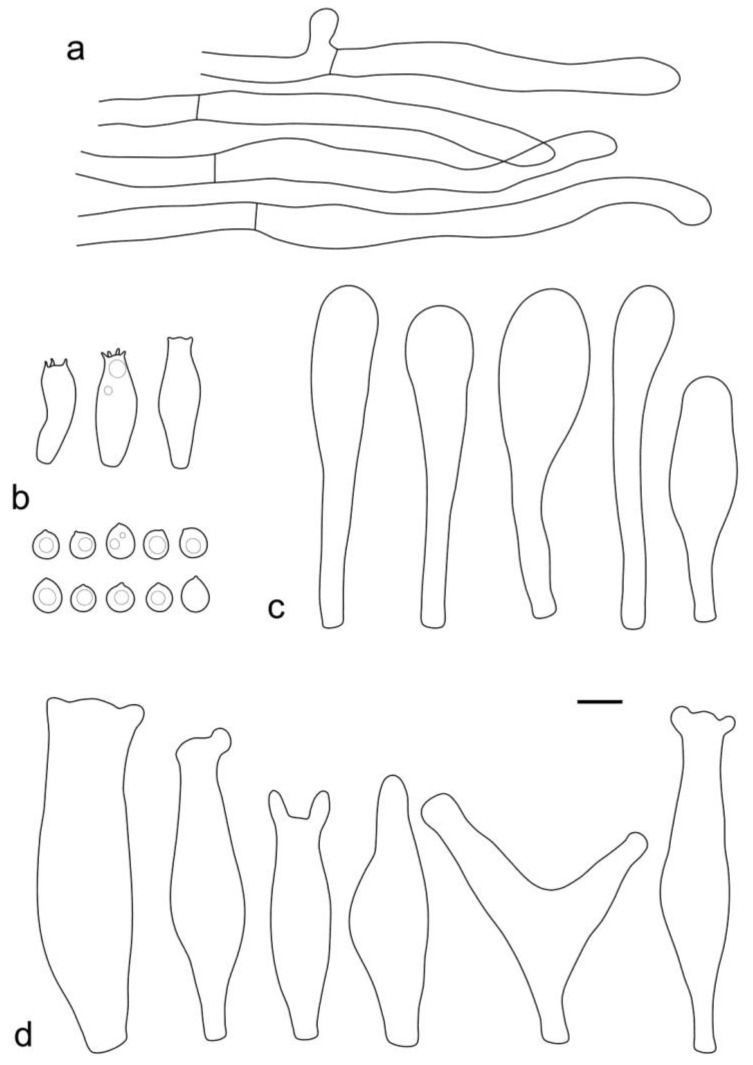
*Pluteus* aff. *septocystidiatus* (LE F-313667) microcharacters: (**a**) pileipellis elements; (**b**) basidia and spores; (**c**) cheilocystidia; (**d**) pleurocystidia. Scale bar 10 μm.

***Pluteus* aff. *septocystidiatus*** (Figure 6 and Figure 8).

Pileus 15–30 mm in diam., when young campanulate, expanding to convex or applanate with slightly concave center; slightly hygrophanous, striate or sulcate at margin; pale brown (RAL 8025) or beige grey (RAL 7006), with darker terra brown (RAL 8025) fibrils; surface radially fibrillose. Lamellae free, moderately distant, ventricose, pink, with concolorous even edges. Stipe 20–40 × 2–4 mm, almost cylindrical or slightly broadened towards base but without bulb, white, shining, glabrous or slightly pruinose at base. Smell indistinct, taste not recorded.

Basidiospores [100/3/1] 6.0–8.2(–9.3) × 5.2–7.0(–7.8) µm (Lav = 7.2, Wav = 6.2, Q = 1.1–1.2(1.3); Qav = 1.2), predominantly broadly ellipsoid or subglobose, highly variable in size, thick-walled. Basidia 19–27 × 7.5–9.5 µm, 2- and 4-spored, broadly clavate. Pleurocystidia 62–79 × 12–24 µm, rather numerous, narrowly to broadly fusiform, sometimes almost cylindrical or bifurcated, with apical formless, obtuse projections, predominantly non-septate, but septate cystidia also present, hyaline, thin- or thick-walled. Cheilocystidia 52–75 × 12.0–20.5 µm, rather numerous, narrowly to broadly clavate, hyaline, thin-walled. Pileipellis a cutis, made up of cylindrical hyphae 7–10 µm wide, with brown intracellular, sometimes incrusted, pigment, thin- or slightly thick-walled. Stipitipellis a cutis of cylindrical, hyaline, slightly thick-walled, 8–12 µm wide hyphae. Caulocystidia absent. Clamp connections absent.

Habitat: in small groups, on rotten wood.

Collection examined: Vietnam, Quang Nam Province, Nam Giang District, La Dee commune, within 4–5 km from the border with Laos along Highway 14D, Song Thanh Nature Reserve, 15°32′10.871″ N, 107°23′16.926″ E, h = 1038 m.a.s.l., lower montane broad-leaved monsoon forest, fallen trunk, on rotten wood, 2 May 2019, col. E.S. Popov (LE F-313667).

Notes: The appearance of the basidiocarps with characteristically concave pilei, the presence of thick-walled pleurocystidia, and basidiospores measuring Lav = 7.2, Wav = 6.2, allow us to treat this collection as *P. septocystidiatus* with a high degree of certainty. However, according to the phylogenetic analysis, the studied Vietnamese collection occupies a rather distant position on the tree (Figure 1), falling clearly into neither *P. septocystidiatus* clade nor the *P. albostipitatus* clade. As emphasized earlier by authors studying *P. septocystidiatus* [25], and as we can conclude from our own experience of studying Vietnamese collections, both taxa have quite a large variability of morphological characteristics and require further study in regard to clarification of taxonomic distinctive features and geographical distribution.

Thus, at present we cannot definitively attribute our specimen to one or another species.

**Figure 9:**
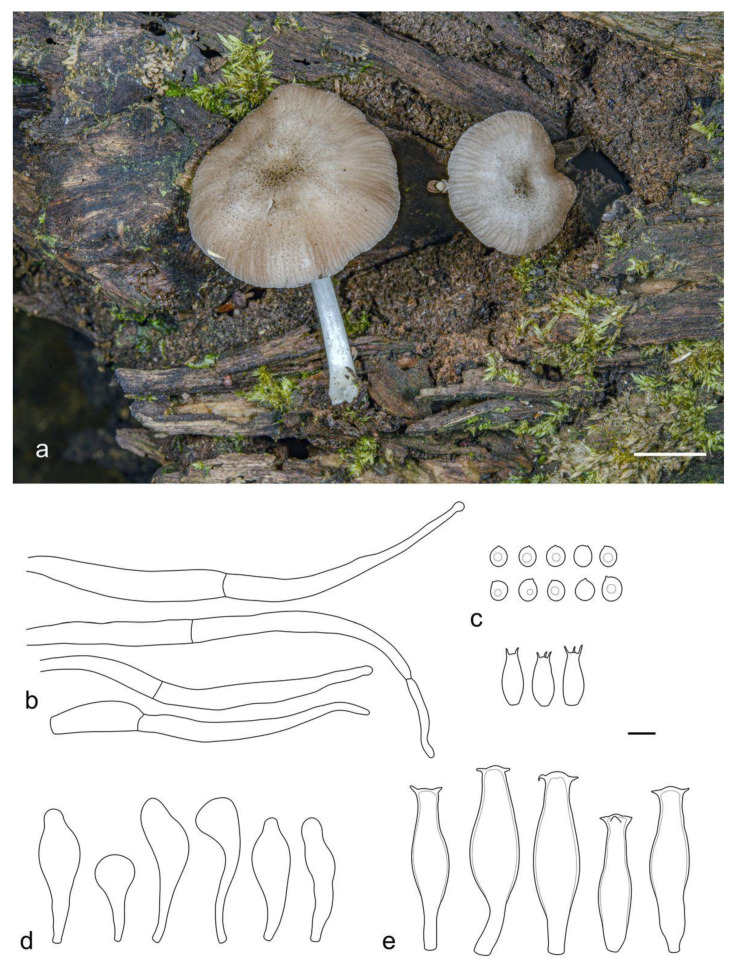
*Pluteus* sp. 1 (LE F-313670): (**a**) basidiocarps in situ; (**b**) pileipellis elements; (**c**) basidia and spores; (**d**) cheilocystidia; (**e**) pleurocystidia. Scale bars 1 cm for (**a**) and 10 μm (for (**b**–**e**)).

***Pluteus* sp. 1** (Figure 9).

Pileus 15–30 mm in diam., at first convex, then plano-convex or applanate with slight central depression; hygrophanous; translucently striate to the half of the radius; beige (RAL 1001), often with pinkish tint; surface squamulose, with scattered brown beige (RAL 1011) or nut brown (RAL 8011) small squamules, densely located at the center. Lamellae free, moderately distant, slightly ventricose, pink, with concolorous even edges. Stipe 20–30 × 2–3 mm, almost cylindrical or slightly broadened towards base but without bulb, white, shining, glabrous. Smell indistinct, taste not recorded.

Basidiospores [60/2/1] 6.0–7.3(–8.3) × (4.7–)5.2–6.7 µm (Lav = 6.8, Wav = 5.7, Q = 1.1–1.3(1.5); Qav = 1.2), subglobose, broadly ellipsoid or oblong, thick-walled. Basidia 21–28 × 7–9 µm, 2- and 4-spored. Pleurocystidia metuloid, 50–65(76) × 12.0–21.5 µm, numerous, narrowly to broadly fusiform, with 2–3(–5) apical, entire and short hooks, hyaline, thick-walled (with wall up to 2 µm). Cheilocystidia 30–50 × 10.0–17.5 µm, abundant, forming a sterile layer at the edge of lamellae, broadly clavate or broadly utriform, hyaline, thin-walled. Pileipellis a cutis, with cylindrical or narrowly fusiform, somewhat ascending terminal elements, 77–120 × 8–11 µm, with brown intracellular pigment, thin- or slightly thick-walled. Stipitipellis a cutis of cylindrical, slightly thick-walled, 7–12 µm wide, hyaline hyphae. Caulocystidia absent. Clamp connections scarce.

Habitat: in small groups, on rotten wood.

Collections examined: Vietnam, Quang Nam Province, Nam Giang District, La Dee commune, within 4–5 km from the border with Laos along Highway 14D, Song Thanh Nature Reserve, 15°33′14.504″ N, 107°23′13.671″ E, h = 1073 m.a.s.l., lower montane broad-leaved monsoon forest, fallen trunk, on rotten wood, 7 May 2019, col. E.S. Popov (LE F-313670, duplicate in VRTC—ESP 269).

Notes: The collection studied is similar to several specimens from Vanuatu on the basis of nrITS sequences. They are all located in the same clade with the highest statistical support (BS = 100% and PP = 1.0). The species identified preliminary as *Pluteus* sp. nov. 1 by J.A. del Rosario in his recent work [26] is characterized by a greyish-brown to hazel appressed-fibrillose, hygrophanous pileus with a white bulbous base stipe, tissues acquiring a faint bluish-grey color when disturbed or handled, subglobose basidiospores with a mean size of 7.3 × 6.6 μm, clavate to utriform cheilocystidia, fusoid to lageniform thick-walled pleurocystidia with apices varying from 2–5 poorly to well-developed hooks/horns, a cutis pileipellis with clavate to somewhat filiform terminal elements, absent caulocystidia, and the presence of clamp connections.

The Vietnamese specimen almost completely fits the description given, except for the tissue turning blue, which we did not observe in fresh basidiocarps. However, we admit such a tissue reaction in our specimen, which may have been very slow and therefore was not noticed by us. Thus, additional research is needed to clarify this feature.

The species is currently in the publication phase (Del Rosario, personal communication), but in his earlier work [26], the author provided a very detailed description of this taxon and a comparison of the collections from Vanuatu with closely related species. Therefore, in this article, we leave our collection unnamed for the time being.

#### 3.2.2. Sect. *Celluloderma*

**Figure 10:**
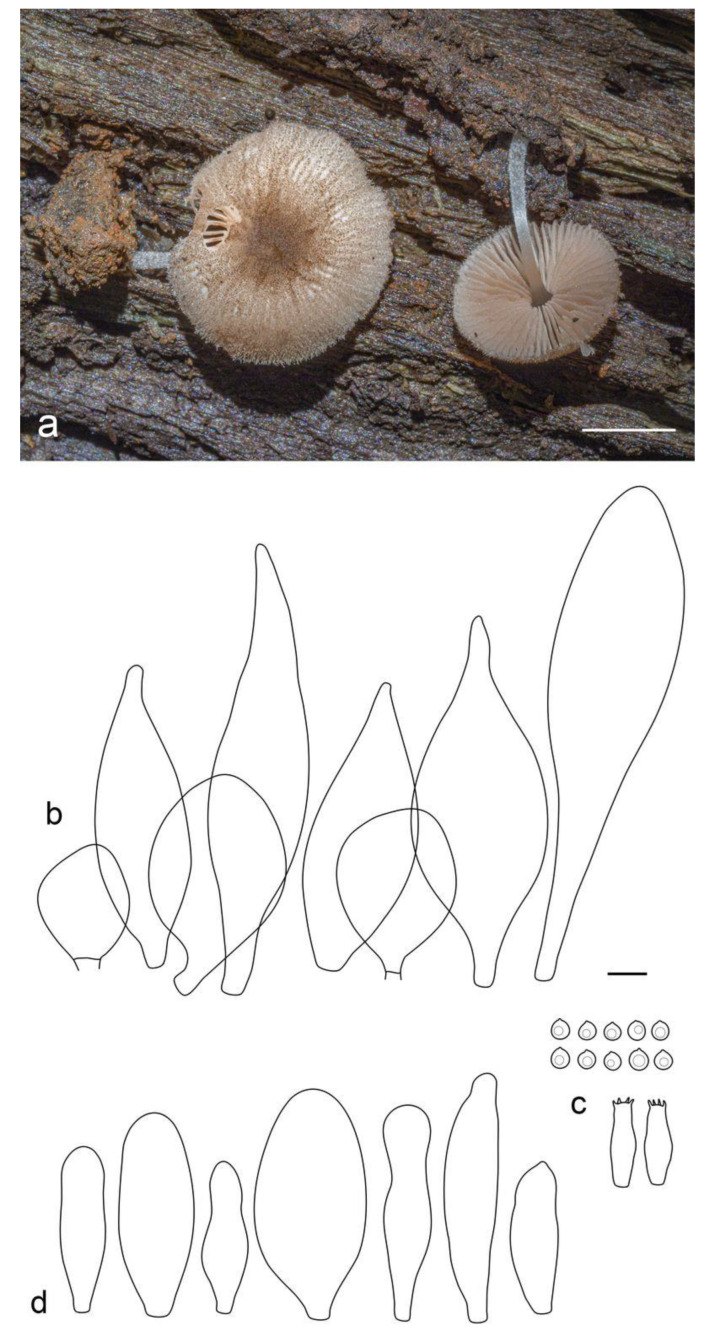
*Pluteus lucidus* holotype (LE F-347426): (**a**) basidiocarps in situ; (**b**) pileipellis elements; (**c**) basidia and spores; (**d**) cheilocystidia. Scale bars 5 mm for (**a**) and 10 μm (for (**b**–**d**)).

***Pluteus lucidus*** E.F. Malysheva, sp. nov. (Figure 10).

MB 848251.

Etymology: the epithet “lucidus” refers to light-colored, whitish and shining stipe.

Diagnosis: differs from *P. seticeps* as it is characterized by the absence of dark brown fibrils on the stipe surface, utriform or lageniform cheilocystidia, and the absence of caulocystidia.

Holotype: Vietnam, Dak Nong Province, Dak Glong District, Ta Dung National Park, south-eastern slope of the Ta Dung Mt, TK 1805, 11°50′34.836″ N, 108°03′40.320″ E, h = 1211 m.a.s.l., evergreen broad-leaved forest (with *Lithocarpus* spp., *Quercus* sp., *Schima* sp., *Dilleniaceae*, *Myristicaceae*, *Acer flabellatum*), on rotten wood, 12 October 2022, col. V.A. Dudka (LE F-347426).

Pileus 7–12 mm in diam., initially convex, becoming plano-convex without umbo; not hygrophanous, striate-sulcate to the center; surface minutely squamulose, granulose at center, brown beige (RAL 1011) or beige brown (RAL 8024), with darker nut brown (RAL 8011) disc; margin slightly incurved, pubescent. Lamellae free, rather distant, slightly ventricose, pink, with concolorous even edges. Stipe 8–15 × 0.7–1.2 mm, almost cylindrical or slightly broadened towards base, but without basal bulb, entirely whitish, covered in the lower half with pellucid sparse hairs. Smell indistinct, taste not recorded.

Basidiospores [70/2/2] 4.0–5.2 × 3.7–5.0 μm (Lav = 4.7, Wav = 4.2, Q = 1.0–1.2; Qav = 1.1), globose or subglobose, thick-walled. Basidia 15–22 × 6.5–9.7 μm, 4-spored. Pleurocystidia absent. Cheilocystidia 37.0–67.5 × (8–)12–20(–31) μm, numerous, utriform or broadly lageniform with short neck and often subcapitate apex, sometimes with small papilla at apex, hyaline, slightly thick-walled. Pileipellis a transition between hymeniderm and epithelium, made up of two types of elements: (1) long broadly fusiform elements with acute apices, 68–157 × 22–36 μm, and (2) broadly clavate or spheropedunculate elements, 25–45(–57) × 14.5–38.0 μm; all types with dark brown, irregularly diffused, intracellular pigment, slightly thick-walled. Stipitipellis a cutis of cylindrical, hyaline, slightly thick-walled, 5–12 μm wide hyphae. Caulocystidia not seen. Clamp connections absent in all tissues.

Habitat: solitary, on dead wood.

Additional collection examined: Vietnam, Dak Nong Province, Dak Glong District, Ta Dung National Park, south-eastern slope of the Ta Dung Mt, TK 1805, 11°50′34.836″ N, 108°03′40.320″ E, h = 1211 m.a.s.l., evergreen broad-leaved forest (with *Lithocarpus* spp., *Quercus* sp., *Schima* sp., *Dilleniaceae*, *Myristicaceae*, *Acer flabellatum*), on rotten wood, 12 October 2022, col. V.A. Dudka (isotype in VRTC—G548).

Notes: *Pluteus lucidus* is characterized by diminutive basidiocarps, dark brown granulose-squamulose pileus, totally white stipe, the absence of pleuro- and caulocystidia, utriform or broadly lageniform cheilocystidia, and mixed pileipellis consisting of two types of elements.

According to macro- and microscopic features, *P. lucidus* most resemble *P. seticeps*, *P. podospileus* and *P. podospilloides*. Recent molecular studies have shown that these species apparently belong to a species complex [2,6], and similar conclusions have been made before [27]. *P. lucidus* can be considered as a part of *P. seticeps* species complex, and it occupies a sister position to the *P. seticeps* clade on the phylogenetic tree (Figure 2). However, *P. lucidus* is distinguished from the latter species by the lack of brown fibrils on stipe and utriform or lageniform cheilocystidia, which are sphaeropedunculate or clavate in *P. seticeps* [27]. Other similar species, *P. podospileus* and *P. podospilloides*, all differ from *P. lucidus* in the possession of pleurocystidia.

**Figure 11:**
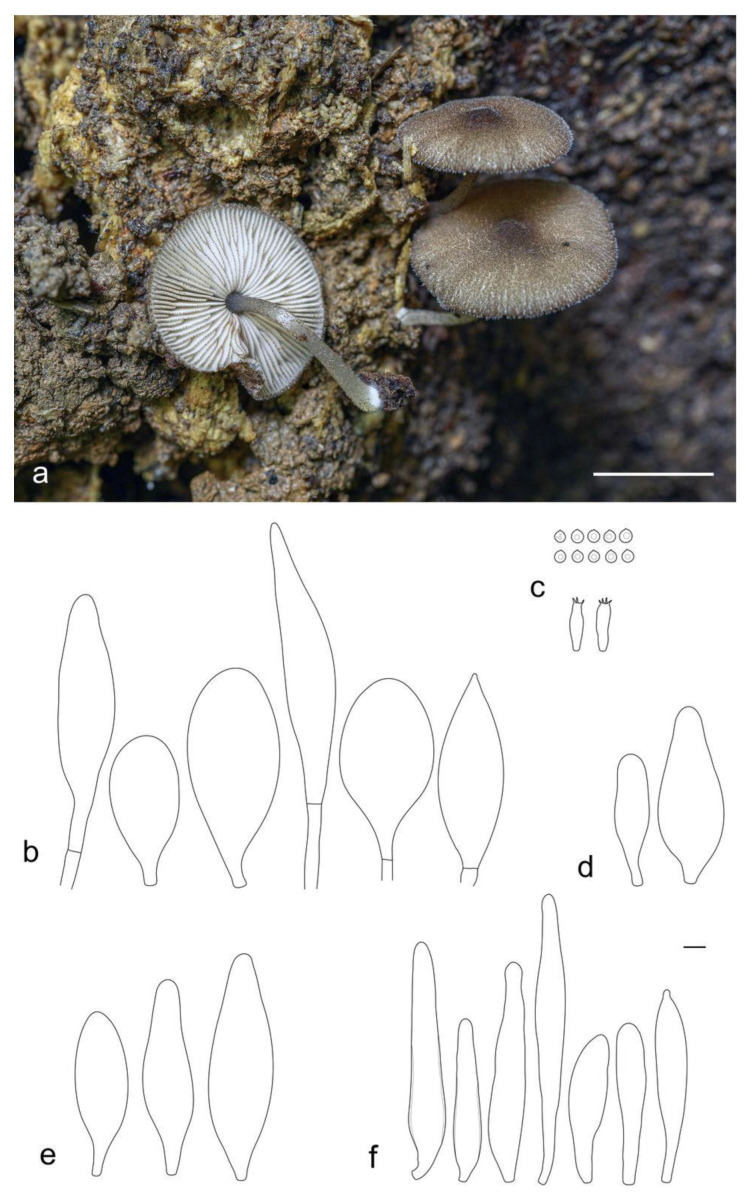
*Pluteus podospilloides* (LE F-347430): (**a**) basidiocarps in situ; (**b**) pileipellis elements; (**c**) basidia and spores; (**d**) pleurocystidia; (**e**) cheilocystidia; (**f**) caulocystidia. Scale bars 1 cm for (**a**) and 10 μm (for (**b**–**f**)).

***Pluteus podospilloides*** E.F. Malysheva et O.V. Morozova, in Malysheva, Malysheva, Alexandrova and Morozova, Phytotaxa 461(2): 100 (2020) (Figure 11)

Pileus 7–15 mm in diam., initially hemispherical, expanding to plano-convex with low broad umbo; not hygrophanous, not striate; surface felty-hairy, dotty floccose-squamulose, covered with minute brown beige (RAL 1011), clay brown (RAL 8003), nut brown (RAL 8011) or beige brown (RAL 8024) squamules together with white short hairs; margin slightly incurved. Lamellae free, crowded, slightly ventricose, yellowish pink, with dark brown fringed edges. Stipe 10–15 × 1–1.7 mm, almost cylindrical or slightly broadened towards base, with small basal bulb, whitish, entirely covered with scattered brown beige (RAL 1011) or clay brown (RAL 8003) squamules. Smell indistinct, taste not recorded.

Basidiospores [120/4/3] 4.1–5.6 × 3.7–5.0 μm (Lav = 4.8, Wav = 4.3, Q = 1.0–1.2; Qav = 1.1), globose or subglobose, thick-walled. Basidia 18.5–24.0 × 5.5–8.0 μm, 4-spored. Pleurocystidia 40–50 × 16–20 μm, scarce to very rare, utriform or broadly lageniform with short neck, with brown intracellular pigment, slightly thick-walled. Cheilocystidia 45–98 × 12.5–29.0(–34) μm, abundant, forming a sterile layer at the edge of lamellae, variable in shape, mostly broadly to narrowly fusiform, lanceolate, more rarely subcylindrical, narrowly utriform or clavate, with dark brown intracellular pigment distributed inside cells in the form of irregular spots, slightly thick-walled. Pileipellis a transition between hymeniderm and epithelium, made up of two types of elements: (1) long narrowly to broadly fusiform elements with obtuse or acute apices, (67–)115–150 × 22–38 μm, and (2) broadly clavate or spheropedunculate elements, (35–)47–82 × 28–45 μm; all types with dark brown, irregularly diffused, intracellular pigment, slightly thick-walled. Stipitipellis a cutis of cylindrical, hyaline, slightly thick-walled, 5–8 μm wide hyphae. Caulocystidia numerous, in bundles, 50–144(–164) × (8.5–)11–19 μm, narrowly fusiform or cylindrical, rarely clavate, with brown intracellular pigment, irregularly diffused throughout the cell, but more densely located at the apex. Clamp connections absent in all tissues.

Habitat: solitary, on dead wood.

Collections examined: Vietnam, Quang Nam Province, Nam Giang District, La Dee commune, within 4–5 km from the border with Laos along Highway 14D, Song Thanh Nature Reserve, 15°34′04.462″ N, 107°23′05.794″ E, h = 1026 m.a.s.l., lower montane broad-leaved monsoon forest, fallen trunk, on rotten wood, 30 April 2019, col. E.S. Popov (LE F-313666, duplicate in VRTC—ESP-004514); Dak Nong Province, Dak Glong District, Ta Dung National Park, south-eastern macroslope of the ridge of the Ta Dung Mt, south-eastern slope of the Ta Dung Mt, TK 1805, 11°31′09.948″ N, 108°25′26.940″ E, h = 1257 m.a.s.l., evergreen broad-leaved forest (with *Lithocarpus* spp., *Quercus* sp., *Schima* sp., *Dilleniaceae*, *Myristicaceae*, *Acer flabellatum*), on rotten stump, 11 October 2022, col. O.V. Morozova (LE F-347430, duplicate in VRTC—94VN22); 11°52′04.080″ N, 108°07′00.912″ E, h = 1241 m.a.s.l., evergreen broad-leaved forest, on rotten wood, 15 October 2022, col. O.V. Morozova (LE F-347431).

Notes: This species was described recently from the territory of Vietnam [2]. Morphologically, it is very similar to *P. podospileus* Sacc. et Cub., except for the presence of relatively smaller basidiocarps, dark brown lamellar edges, colored pleuro- and cheilocystidia, and smaller basidiospores (vs. 5.3–6.6 × 4.4–5.3 µm [27] or 5.5–7.5(8.0) × (4.0)4.5–6.0 µm [5] in *P. podospileus*). A detailed comparison of *P. podospilloides* with closely related species can be found in our previous paper [2].

The collections analyzed in this study are new findings of this species in Vietnam. Moreover, each time the specimens were found on rotten wood in the mountainous regions. Although the ecology and geographical distribution of the species still need to be clarified, we can already conclude that it is quite common and widespread in the territory of Vietnam.

**Figure 12:**
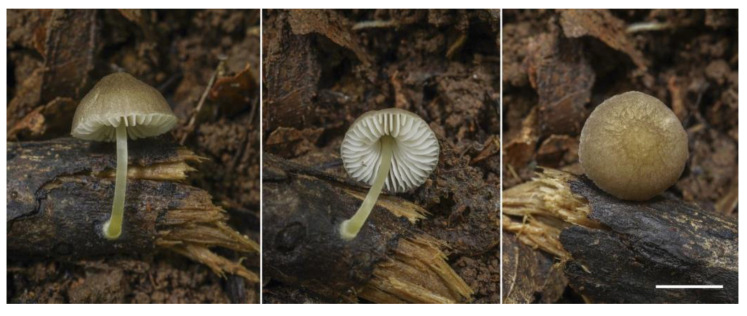
Basidiocarps of *Pluteus* aff. *pauperculus* (LE F-347434) in situ. Scale bar 5 mm.

**Figure 13:**
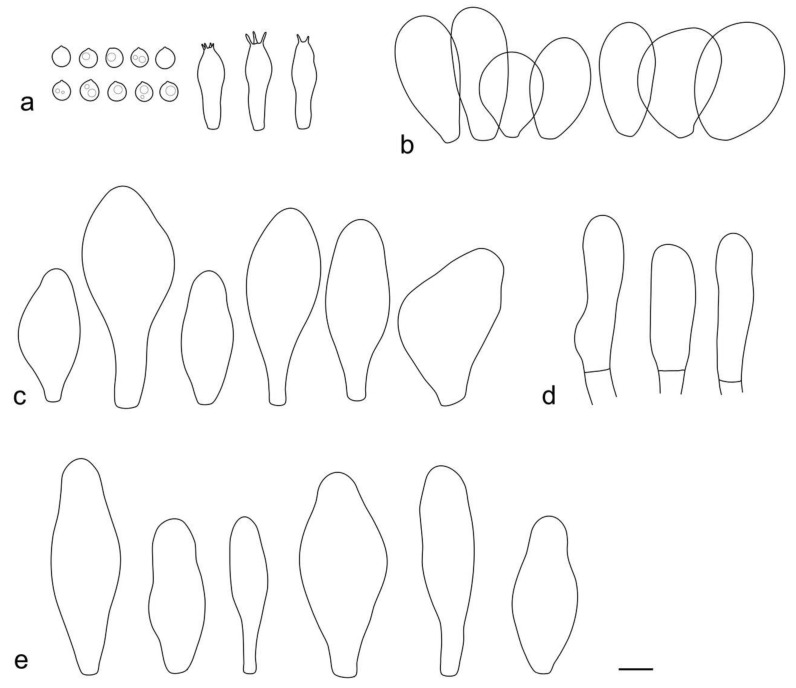
*Pluteus* aff. *pauperculus* (LE F-347434) microcharacters: (**a**) basidia and spores; (**b**) pileipellis elements; (**c**) cheilocystidia; (**d**) caulocystidia; (**e**) pleurocystidia. Scale bar 10 μm.

***Pluteus* aff. *pauperculus*** (Figure 12 and Figure 13).

Pileus 6–8 mm in diam., initially hemispherical or campanulate, becoming convex with broad umbo; not hygrophanous; surface minutely granulose, strongly venose across the entire surface, with a distinct reticulate pattern in the center, sometimes cracked near edge, brown beige (RAL 1011) with honey yellow tint (RAL 1005), ochre brown (RAL 8001), with clay brown (RAL 8003) center; margin not striate, slightly serrated. Lamellae free, distant, ventricose, whitish, becoming pink, with concolorous even edges. Stipe 10–12 × 0.5–1.0 mm, almost cylindrical or slightly broadened towards swollen or subbulbose base, in the upper half pale sulfur yellow (RAL 1016), at the bottom zinc yellow (RAL 1018) or colza yellow (RAL 1021), smooth or longitudinally fibrillose. Smell indistinct, taste not recorded.

Basidiospores [100/2/2] 5.0–6.5 × 4.5–6.2 μm (Lav = 5.8, Wav = 5.3, Q = 1.0–1.2; Qav = 1.1), globose or subglobose, thick-walled. Basidia 21–27 × 7.0–8.5 μm, 2- and 4-spored, clavate. Pleurocystidia 48–68 × 12–21(–28.5) μm, scattered, narrowly to broadly utriform, hyaline, thin- or slightly thick-walled. Cheilocystidia 38.5–60.0(–67.5) × 16.5–32.5 μm, numerous, utriform or broadly fusiform, hyaline, thin-walled. Pileipellis a hymeniderm, made up of broadly clavate or spheropedunculate elements, 30–45 × 16.5–25.0 μm, with dark grey-brown intracellular pigment, slightly thick-walled. Stipitipellis a cutis of cylindrical, hyaline, slightly thick-walled, 5–9 μm wide hyphae. Caulocystidia rare, (42–)48–70 × 10–13 μm, in bundles, cylindrical or narrowly clavate. Clamp connections absent in all tissues.

Habitat: solitary, on dead wood.

Collections examined: Vietnam, Dak Nong Province, Dak Glong District, Ta Dung National Park, south-eastern slope of the Ta Dung Mt, TK 1805, 11°50′34.584″ N, 108°03′43.344″ E, h = 1200 m.a.s.l., evergreen broad-leaved forest (with *Lithocarpus* spp., *Quercus* sp., *Schima* sp., *Dilleniaceae*), on rotten twig, 12 October 2022, col. O.V. Morozova (LE F-347433); Vietnam, Dak Nong Province, Dak Glong District, Ta Dung National Park, south-eastern slope of the Ta Dung Mt, TK 1805, 11°52′04.080″ N, 108°07′00.912″ E, h = 1241 m.a.s.l., evergreen broad-leaved forest (with *Lithocarpus* spp., *Quercus* sp., *Schima* sp., *Dilleniaceae*, *Myristicaceae*, *Acer flabellatum*), on rotting branch, 10 October 2022, col. O.V. Morozova, T.H.G. Pham (LE F-347434).

Notes: The studied Vietnamese collections match *P. pauperculus* in almost all characters, except that they are considerably more diminutive than the New Zealand and Western Australian collections on which the species is based [24]. In addition, our specimens have caulocystidia, but they are rare.

From descriptions of modern collections of the species currently available on the Internet (http://iucn.ekoo.se/iucn/species_view/531297/; https://www.gbif.org/es/species/5241379, accessed on 1 March 2023), it is obvious that the variation in macro-characters and substrate preferences may indicate that in this case we are dealing not with a single taxon, but with a complex of species. An additional confirmation of this assumption is the phylogenetic analysis performed (Figure 2). There are two nrITS sequences of this taxon in the GenBank database (MN738621, MN738636), and our specimens do not form a single clade with either of them. Moreover, all sequences of “*P. pauperculus*” occupy an independent position on the tree, close to species from the /romellii group (*P. vellingae* Ševčíková, Kaygusuz, G. Muñoz, Lebeuf et S. D. Russell, *P. parvicarpus* E.F. Malysheva, *P. siccus* E.F. Malysheva, *P. aureovenatus* Menolli et Capelari and *P. sublaevigatus* (Singer) Menolli et Capelari), but are not included in any clade. Hence, further research is required to determine the taxonomic and phylogenetic status of distinct populations of this species, their geographic ranges and habitat preferences.

#### 3.2.3. Sect. *Hispidoderma*

**Figure 14:**
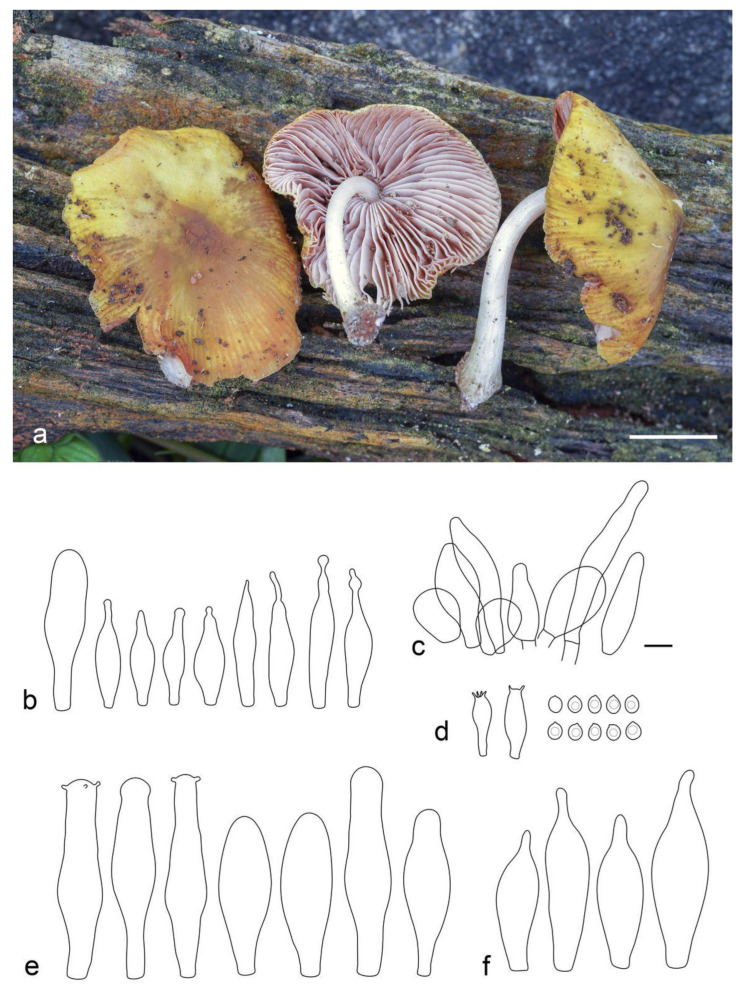
*Pluteus chrysaegis* (LE F-347436): (**a**) basidiocarps in situ; (**b**) cheilocystidia; (**c**) pileipellis elements; (**d**) basidia and spores; (**e**) pleurocystidia; (**f**) caulocystidia. Scale bars 1 cm for (**a**) and 10 μm (for (**b**–**f**)).

***Pluteus chrysaegis*** (Berk. et Broome) Petch, Ann. R. bot. Gdns Peradeniya 5(4): 271 (1912) (Figure 14).

Pileus 25–30 mm in diam., initially broadly campanulate, becoming plano-convex with broad umbo; hygrophanous, translucently striate to the half of the radius; surface smooth, wrinkled at the center, brightly colored—zinc yellow (RAL 1018), colza yellow (RAL 1021), with darker yellow orange (RAL 2000) center; margin even, slightly incurved. Lamellae free, rather crowded, slightly ventricose, pink, with concolorous even edges. Stipe 15–25 × 2.0–3.5 mm, almost cylindrical or slightly broadened towards swollen base, whitish, with grey beige (RAL 1019) fibrils at base, smooth or longitudinally fibrillose. Smell indistinct, taste not recorded.

Basidiospores [60/2/1] 5.2–6.7 × 4.2–5.6 μm (Lav = 5.8, Wav = 4.9, Q = 1.1–1.3(–1.5); Qav = 1.2), broadly ellipsoid or ovoid, rarely subglobose, thick-walled. Basidia 18–25 × 6–8 μm, 2- and 4-spored, clavate. Pleurocystidia 52.5–74.0 × 14–19 μm, scattered, narrowly to broadly lageniform, utriform or broadly fusiform, some with short apical projections, hyaline, thin- or thick-walled. Cheilocystidia 32–46 × 9–15 μm, numerous, narrowly to broadly fusiform, with long appendage toward apex or with capitate apex, hyaline, thin-walled. Pileipellis a hymeniderm, made up of broadly clavate elements, 20–30 × 10–20 μm, intermixed with fusiform elements, 30–60 × 12–18 μm, colorless, slightly thick-walled. Stipitipellis a cutis of cylindrical, hyaline, slightly thick-walled, 7–12 μm wide hyphae. Caulocystidia 40–70 × 15–20 μm, in bundles, similar in shape to cheilocystidia, thick-walled. Clamp connections absent in all tissues.

Habitat: in small groups, on dead wood.

Collections examined: Vietnam, Dak Nong Province, Dak Glong District, Ta Dung National Park, south-eastern macroslope of the ridge of the Ta Dung Mt, eastern slope of the M’neun Tchirke Mt, TK 1794, 11°52′38.604″ N, 108°05′31.020″ E, h = 1100 m.a.s.l., trail in the coffee plantation, on a live tree, 9 October 2022, col. E.S. Popov (LE F-347436, duplicate in VRTC—76VN22).

Notes: *Pluteus chrysaegis*, originally described from Sri Lanka, is characterized by the combination of golden-yellow basidiocarps with brown rugulose center, subglobose to globose basidiospores, lageniform to fusoid cheilocystidia with long appendages, abundant fusiform and thick-walled pleurocystidia, and a pileipellis composed of clavate cells intermixed with fusiform pileocystidia [28,29,30,31].

While comparing *P. chrysaegis* with a very similar species, *P. conizatus* var. *africanus* E. Horak, originally described from Equatorial Africa [32], which was previously differentiated from *P. chrysaegis* based on the thickened wall of the hymenial cystidia and the size of pileipellis elements, Pradeep et al. [30] concluded that these characteristics were not reliable for separating the two taxa, because they were highly variable even in one specimen. Based on a detailed morphological study of the specimens of both taxa, including the type collection of *P. conizatus* var. *africanus*, the aforementioned authors began to consider the latter as synonymous to *P. chrysaegis*.

*Pluteus chrysaegis*, in its new conception, is now known from India [28,29,30], Equatorial and West Africa [31,32], the United States and China [33], and from Vietnam. These data indicate that this species has a very wide geographic distribution, correlating with a wide range of variability in micromorphological characteristics. Thus, the Vietnamese specimens we studied were characterized, in contrast to previous observations, by predominantly ellipsoid basidiospores, cheilocystidia with subglobose and globose apexes, the presence of apical projections in pleurocystidia, as well as the presence of quite numerous caulocystidia on the stipe surface. The phylogenetic analysis (Figure 3) showed that the studied collection belonged to *P. chrysaegis* clade with high statistical support (PP = 1.00, BS = 96%).

**Figure 15:**
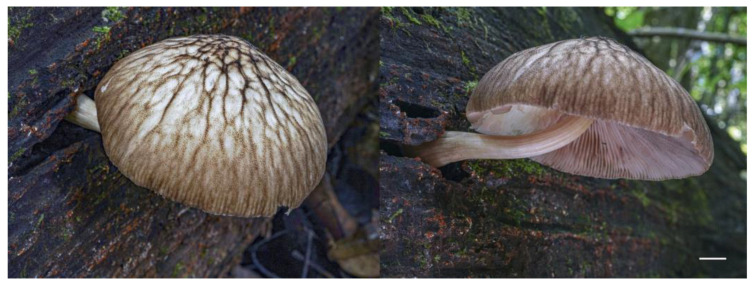
Basidiocarp of *Pluteus ornatus* holotype (LE F-347437) in situ. Scale bar 1 cm.

**Figure 16:**
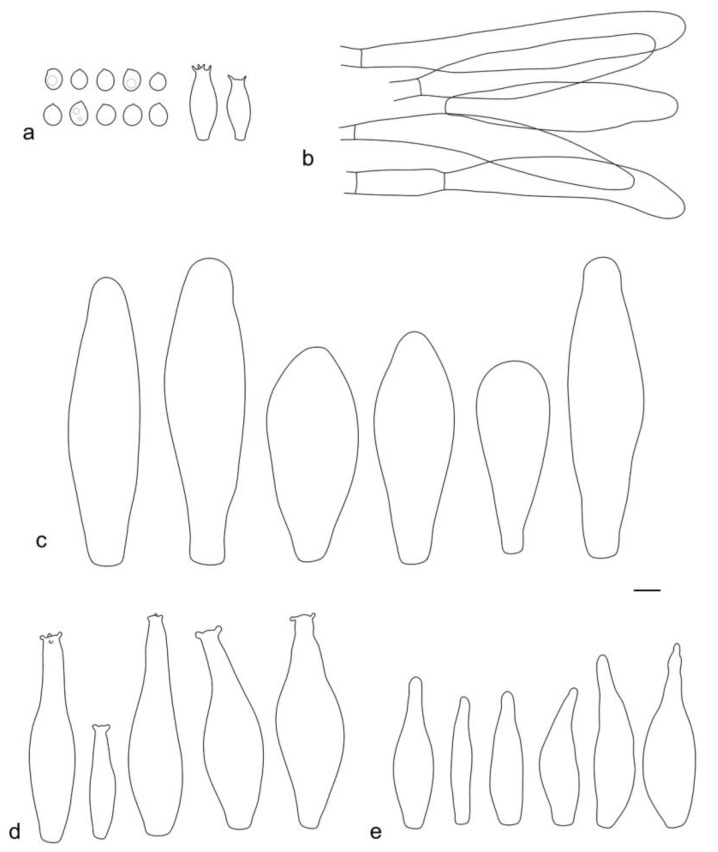
*Pluteus ornatus* holotype (LE F-347437) microcharacters: (**a**) basidia and spores; (**b**) pileipellis elements; (**c**) cheilocystidia; (**d**) pleurocystidia; (**e**) caulocystidia. Scale bar 10 μm.

***Pluteus ornatus*** E.F. Malysheva, sp. nov. (Figure 15 and Figure 16).

MB 848252.

Etymology: the epithet, from the Latin word “ornatus” (adorned), is proposed to indicate a remarkable pileus ornamentation with squamules.

Diagnosis: the species is characterized by its large stout basidiocarps with densely squamulose, dark brown pileus, lamellae with brown edges, pale colored stipe, ellipsoid basidiospores with mean 7.7 × 6.3 μm, lageniform pleurocystidia with apical excrescences, colored clavate cheilocystidia, and pileipellis as trichohymeniderm.

Holotype: Vietnam, Dak Nong Province, Dak Glong District, Ta Dung National Park, M’neun Tchirke Mt, TK 1794 and TK 1795, 11°53′59.604″ N, 108°04′57.396″ E, h = 1300 m.a.s.l., secondary evergreen broad-leaved forest (with *Fagaceae*, *Dilleniaceae*, *Theaceae*, with significant participation of bamboos, *Calamus* sp., *Rubus* sp.), on rotten wood, 19 October 2022, col. V.A. Dudka (LE F-347437).

Pileus 80–100 mm in diam., hemispherical at first, then convex, with low broad umbo; not hygrophanous; surface densely squamulose, with erect tapered squamules, densely located at the center and forming pattern of veins radiating in a network from the center to the margin, showing whitish background between; squamules signal brown (RAL 8002), copper brown (RAL 8004) or fawn brown (RAL 8007) at margind, and mahogany brown (RAL 8016), chocolate brown (RAL 8017) or grey brown (RAL 8019) at center; margin even, striate. Lamellae free, moderately crowded, ventricose, pink, with brown, serrulate edges. Stipe 70–90 × 6–13 mm, thickened downwards, longitudinally fibrillose, shiny, beige (RAL 1001) or sand yellow (RAL 1002). Smell indistinct, taste not recorded.

Basidiospores [80/2/1] 6.5–9.5 × 5.3–7.7 µm (Lav = 7.7, Wav = 6.3, Q = 1.1–1.3(–1.4); Qav = 1.2), predominantly ellipsoid and broadly ellipsoid, occasionally subglobose or cylindrical, thick-walled. Basidia 25–30 × 9–11 µm, 4-spored, clavate. Pleurocystidia (45–)52–82 × (8.5–)14–22(–27) µm, scattered, broadly to narrowly lageniform or broadly fusiform, with 2–4 short apical excrescences, hyaline, thin-walled. Cheilocystidia 47–110 × 17–33(–42) µm, abundant, forming a sterile layer at the edge of lamellae, fusiform or clavate, with brown intracellular pigment, thin- or slightly thick-walled. Pileipellis a trichohymeniderm, consisting of narrowly to broadly fusiform terminal elements with tapering or obtuse apexes, 85–117 × 15–22 µm, with yellow-brown intracellular pigment, slightly thick-walled. Stipitipellis a cutis of hyaline cylindrical hyphae 8–15 µm wide. Caulocystidia 46–70 × 9–20 µm, numerous, in clusters, narrowly fusiform or narrowly lageniform, often with acute apex, with brownish intracellular pigment, slightly thick-walled. Clamp connections absent in all tissues.

Habitat: solitary, on rotten wood.

Additional collection examined: Vietnam, Dak Nong Province, Dak Glong District, Ta Dung National Park, M’neun Tchirke Mt, TK 1794 and TK 1795, 11°53′59.604″ N, 108°04′57.396″ E, h = 1300 m.a.s.l., secondary evergreen broad-leaved forest (with *Fagaceae*, *Dilleniaceae*, *Theaceae*, with significant participation of bamboos, *Calamus* sp., *Rubus* sp.), on rotten wood, 19 October 2022, col. V.A. Dudka (isotype in VRTC—182VN22).

Notes: *Pluteus ornatus* is morphologically most similar to known species *P. umbrosus* (Pers.) P. Kumm. and *P. umbrosoides* E.F. Malysheva, both distributed in Eurasia. However, detailed morphological studies indicate that *P. ornatus* can be distinguished from *P. umbrosus* by its smooth, not squamulose stipe, larger basidiospores (vs. 5.5–6.5 × 4.0–5.0 µm, [5]), and smaller terminal elements of pileipellis. *P. umbrosoides* differs in lamellae without brown edges, capitate pleurocystidia and smaller basidiospores [22]. Our phylogenetic analyses indicate that all the three species form well-supported distinct clades (Figure 3). The nrITS sequence of the new species is grouped together with one designated as *P.* aff. *fernandezianus* from Brazil (OM060370, [34]). However, the latter species, originally described from Chile [35], differs significantly from *P. ornatus* in its very small basidiocarps (with pileus not exceeding 2 cm), smaller and differently shaped pleurocystidia, and shorter elements of pileipellis [34,35]. A comparison of the structure of the nrITS sequences also showed their strong difference from each other (see phylogeny section).

**Figure 17:**
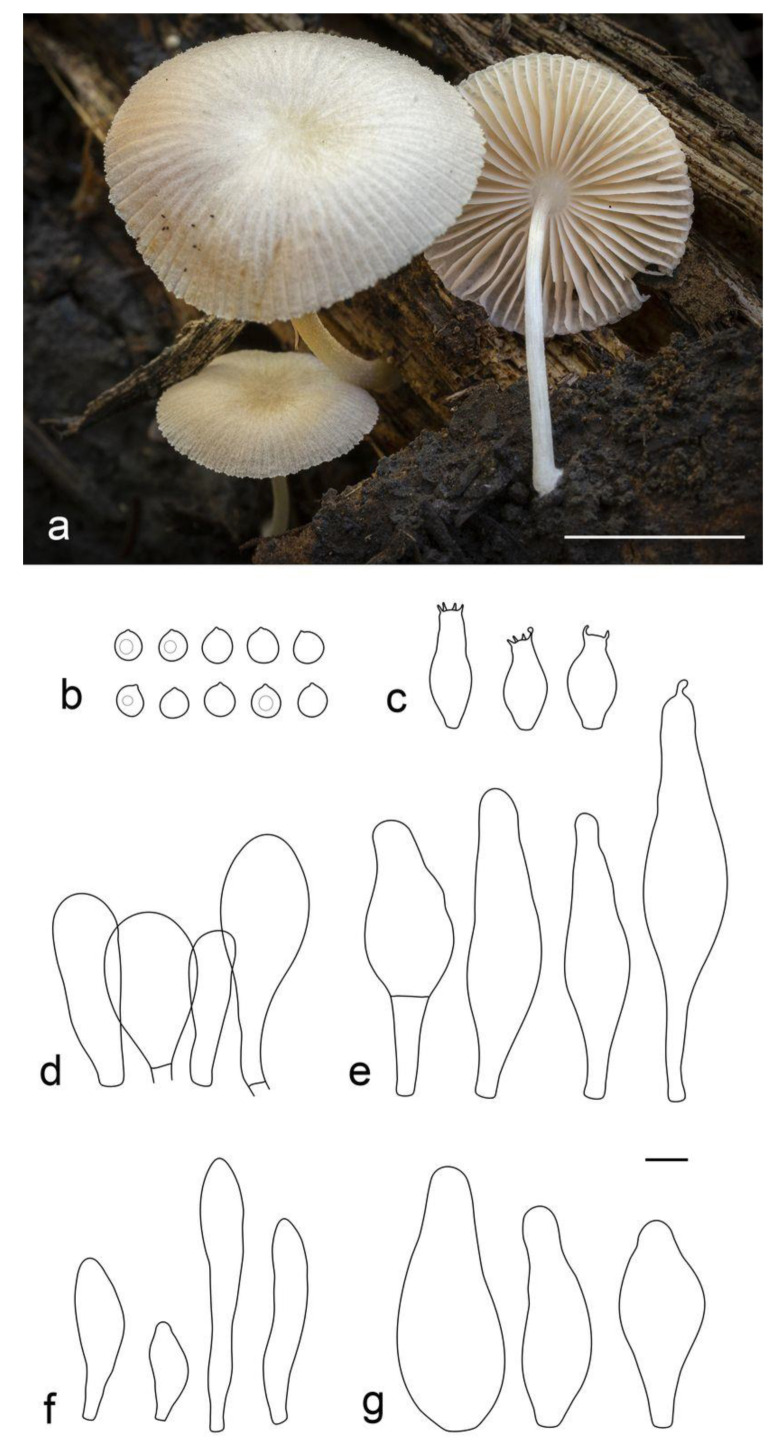
*Pluteus semibulbosus* (LE F-313653): (**a**) basidiocarps in situ; (**b**) spores; (**c**) basidia; (**d**) pileipellis elements; (**e**) pleurocystidia; (**f**) caulocystidia; (**g**) cheilocystidia. Scale bars 1 cm for (**a**) and 10 μm (for (**b**–**g**)).

***Pluteus semibulbosus*** (Lasch) Quél., Mém. Soc. Émul. Montbéliard, Sér. 2 5: 543 (1875) (Figure 17).

Pileus 8–20 mm in diam., convex, becoming plano-convex with concave center; weakly hygrophanous, translucently striate-sulcate to the center; surface minutely granulose, oyster white (RAL 1013), light ivory (RAL 1015), with darker beige (RAL 1001) center; margin serrulate. Lamellae free, distant, slightly ventricose, pink, with concolorous even edges. Stipe 20–25 × 1.5–2.0 mm, almost cylindrical or broadened towards slightly swollen base, whitish or light ivory (RAL 1015), smooth or longitudinally fibrillose. Smell indistinct, taste not recorded.

Basidiospores [65/2/1] 6.3–8.4 × 5.8–7.3 μm (Lav = 7.5, Wav = 6.5, Q = 1.1–1.2; Qav = 1.1), broadly ellipsoid or ovoid, rarely subglobose, thick-walled. Basidia 20–28 × 10.5–12.0 μm, 2- and 4-spored, broadly clavate. Pleurocystidia 60–85(–100) × 13.5–22.0 μm, scattered, narrowly to broadly lageniform, some with short apical projection, hyaline, thin- or thick-walled. Cheilocystidia 43–64 × 17.5–26.5 μm, scattered, broadly lageniform or clavate, thin-walled. Pileipellis a hymeniderm, made up of broadly clavate elements, 45–70 × 17–25 μm, colourless, slightly thick-walled. Stipitipellis a cutis of cylindrical, hyaline, slightly thick-walled, 7–10 μm wide hyphae. Caulocystidia 21.5–63.5 × 8.5–12.0 μm, in bundles, narrowly to broadly fusiform, slightly thick-walled. Clamp connections absent in all tissues.

Habitat: in small group, on dead wood.

Collection examined: Vietnam, Dong Nai Province, Tan Phu District, Cat Tien National Park, 4.5 km to NW from the main reserve station, 011°26′31.530″ N, 107°23′33.750″ E, h = 145 m a.s.l., bottom of a temporary drying stream, polydominant monsoon forest with palms (*Livistona* sp., *Calamus* sp.), bamboo and lianas, on very rotten wooden chips and dust, 19 Feb. 2021, col. A.A. Kiyashko (LE F-313653).

Notes: Originally and based on studies by many authors, *P. semibulbosus* was characterized as a small whitish fungus with a sulcate pileus and pubescent stipe with a distinctly bulbous base [36,37,38]. The complex microscopic characters (pileipellis structure, spore size, shape of hymenial cystidia and caulocystidia) makes the species close to *P. plautus*. It has recently been shown [6,39] that the broad morphological concept of *P. plautus* proposed by Vellinga and Schreurs [40] is not supported by molecular data, and it is obvious that the taxa considered synonymous by these authors (*P. semibulbosus* (Lasch) Gillet and *P. granulatus* Bres.) are different species. Further studies of this group need to be carried out to establish how many taxa can be recognized within the species complex on the basis of morphological and molecular data. Therefore, for the time being, we apply the broad species concept for taxonomic identification of the studied specimen.

**Figure 18:**
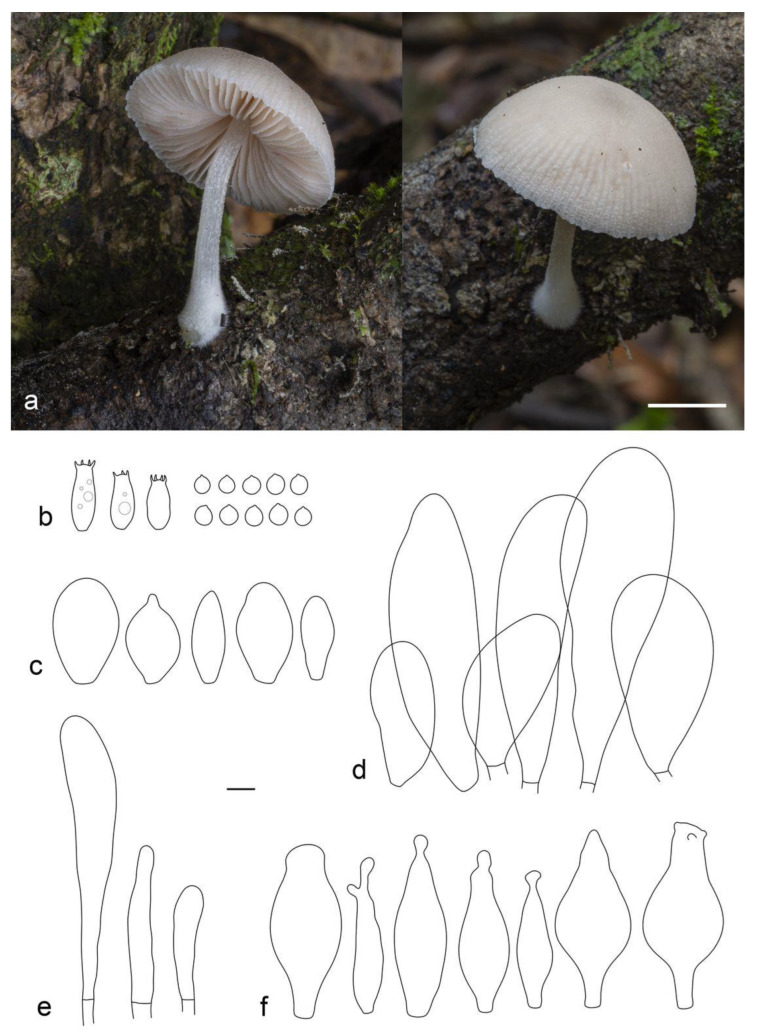
*Pluteus subroseus* holotype (LE F-347429): (**a**) basidiocarps in situ; (**b**) basidia and spores; (**c**) cheilocystidia; (**d**) pileipellis elements; (**e**) caulocystidia; (**f**) pleurocystidia. Scale bars 1 cm for (**a**) and 10 μm (for (**b**–**f**)).

***Pluteus subroseus*** E.F. Malysheva, sp. nov. (Figure 18).

MB 848253.

Etymology: the specific epithet refers to the color of pileus.

Diagnosis: differs from *P. albidus* by pink color of pileus, hymeniderm-type pileipellis and pleurocystidia shape.

Holotype: Vietnam, Dak Nong Province, Dak Glong District, Ta Dung National Park, south-eastern slope of the Ta Dung Mt, 11°51′27.072″ N, 108°03′52.416″ E, h = 1422 m.a.s.l., evergreen broad-leaved forest (with *Lithocarpus* spp., *Quercus* sp., *Schima* sp., *Dilleniaceae*, *Myristicaceae*, *Acer flabellatum*), on decaying wood, 11 October 2022, col. V.A. Dudka (LE F-347429).

Pileus 15–30 mm in diam., when young hemispherical or convex, expanding to plano-convex, without umbo but with slightly concave center; hygrophanous, translucently striate-sulcate to the half of the radius; surface entirely granulose-pruinose, cream (RAL 9001), light ivory (RAL 1015), with darker pale beige red (RAL 3012) center; margin uneven, serrated. Lamellae free, rather crowded, ventricose, pink, with concolorous, serrulate edges. Stipe 20–35 × 2.5–3.5 mm, cylindrical, with subbulbous or distinctly bulbous base, pellucid or whitish, pruinose and fibrillose-striate, with white basal pubescence. Smell indistinct, taste not recorded.

Basidiospores [90/3/1] 6.0–8.6 × 5.5–6.7(–7.5) µm (Lav = 6.9, Wav = 6.2, Q = 1.1–1.2(–1.3); Qav = 1.1), broadly ellipsoid or subglobose, thick-walled. Basidia 19–26 × 8–9 µm, 4-spored. Pleurocystidia 56–85 × 14–30 µm, scattered, inflated fusiform or utriform, with one or several short apical projections, hyaline, thin-walled. Cheilocystidia 33–45 × 12–25 µm, rather numerous, predominantly clavate or utriform, rarely broadly fusiform, some with apical papilla, hyaline, thin-walled. Pileipellis a hymeniderm, made up of clavate or broadly fusiform elements, 55–115(–146) × 23–40 µm, colorless, slightly thick-walled. Stipitipellis a cutis of cylindrical, thick-walled and incrusted, 6–12 µm wide hyphae. Caulocystidia 47–110 × 9–19 µm, in bundles, cylindrical, narrowly clavate or narrowly fusiform, hyaline, slightly thick-walled. Clamp connections absent in all tissues.

Habitat: in small group, on decaying wood.

Additional collection examined: Vietnam, Dak Nong Province, Dak Glong District, Ta Dung National Park, south-eastern slope of the Ta Dung Mt, 11°51′27.072″ N, 108°03′52.416″ E, h = 1422 m.a.s.l., evergreen broad-leaved forest (with *Lithocarpus* spp., *Quercus* sp., *Schima* sp., *Dilleniaceae*, *Myristicaceae*, *Acer flabellatum*), on rotten wood, 11 October 2022, col. V.A. Dudka (isotype in VRTC—G538).

Notes: *Pluteus subroseus* is characterized by rather small basidiocarps with convex, pinkish, radially sulcate and granulose-pruinose pileus, broad lamellae, subbulbous to bulbous stipe, subglobose basidiospores with mean 6.9 × 6.2 μm, cheilocystidia variable in shape, fusiform or utriform pleurocystidia with noticeable apical projections, hymeniform pileipellis, long clavate or fusiform caulocystidia, an absence of clamp connections, and growth on decaying wood.

The closest neighbor to *P. subroseus* nrITS sequence in the molecular phylogenetic tree (Figure 3) is the collection of *P. albidus* from São Tomé and Príncipe (MG968798, [31]), grouped with *P. subroseus* with strong support (PP = 1.00, BS = 86%). However, *P. albidus*, originally described from the DR Congo [41], morphologically differs significantly from our new species by its pure white, glabrous pileus, smaller pleurocystidia without apical projections and cutis-like pileipellis.

Two nrITS sequences (MN738654 and MN738677), labeled as *P. decoloratus* E. Horak from New Zealand, form a highly supported close sister clade with the new species and *P. albidus* (Figure 3). *P. decoloratus*, originally described from New Zealand [24], differs in trichoderm pileipellis with much longer terminal cells, shape of pleurocystidia and the absence of caulocystidia.

*Pluteus subroseus* is macroscopically almost indistinguishable from the species of *P. plautus*–*P. semibulbosus* complex but can be separated by the characteristic shape of pleurocystidia and distinctive nrITS sequences.

**Figure 19:**
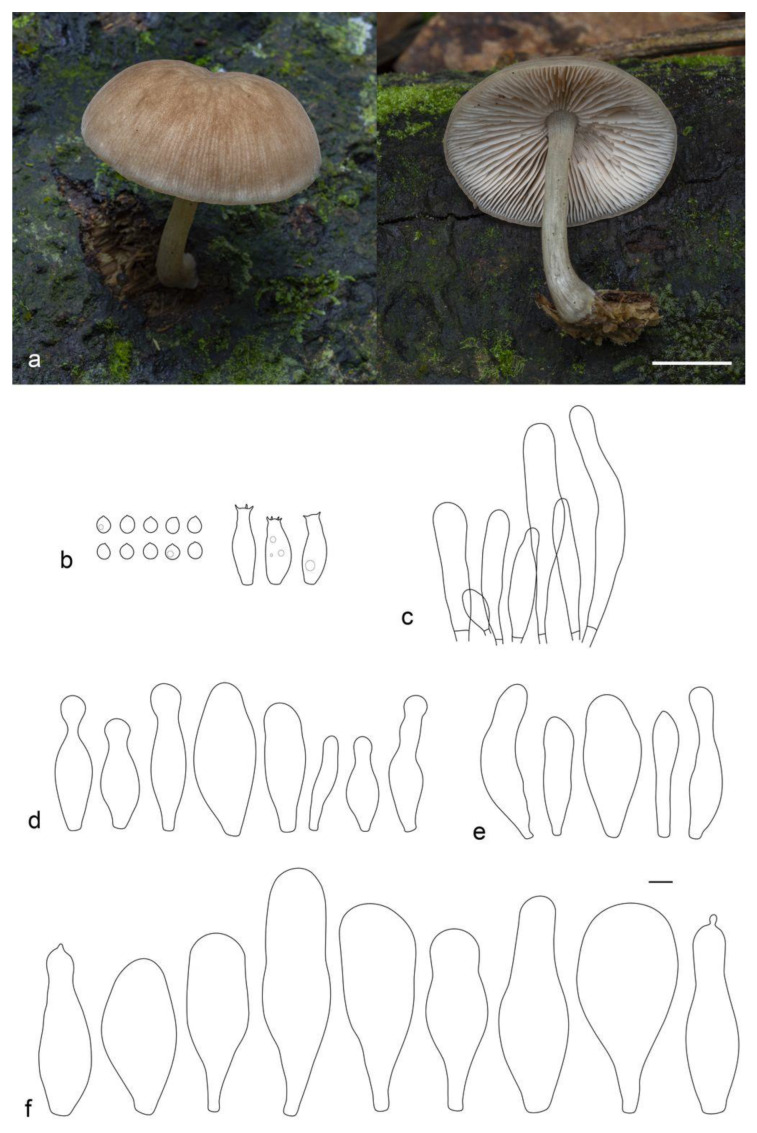
*Pluteus* cf. *velutinus* (LE F-347428): (**a**) basidiocarp in situ; (**b**) basidia and spores; (**c**) pileipellis elements; (**d**) cheilocystidia; (**e**) caulocystidia; (**f**) pleurocystidia. Scale bars 1 cm for (**a**) and 10 μm (for (**b**–**f**)).

***Pluteus* cf. *velutinus*** (Figure 19).

Pileus 15–30 mm in diam., when young hemispherical or convex, expanding to plano-convex, with indistinct umbo or concave center; hygrophanous, translucently striate to the half of the radius; surface entirely granulose-pruinose or slightly velvety, brown beige (RAL 1011), ochre brown (RAL 8001) or orange brown (RAL 8023). Lamellae free, crowded, slightly ventricose, greyish pink or pink, with concolorous even edges. Stipe 15–35 × 1.5–4 mm, cylindrical, with subbulbous or bulbous base, light ivory (RAL 1015), with grey beige (RAL 1019) or ochre brown (RAL 8001) fibrils at lower part, pruinose and fibrillose-striate. Smell indistinct, taste not recorded.

Basidiospores [90/3/2] 5.5–7.6 × (4.6–)5.0–6.0(–6.5) µm (Lav = 6.5, Wav = 5.5, Q = 1.1–1.2; Qav = 1.1), broadly ellipsoid, ovoid or subglobose, thick-walled. Basidia 17–28 × 5.5–9 µm, 2- and 4-spored. Pleurocystidia 40–95(–105) × 11–30 µm, rather numerous, varying in shape in different specimens: broadly fusiform or lageniform, often tapering at apex or with a single apical protuberance; or broadly utriform, broadly clavate or spatuliform; with yellow-brown intracellular pigment, thin- or slightly thick-walled. Cheilocystidia 37–55(60) × 10.5–19.0 µm, numerous, utriform or lageniform with short neck and predominantly subcapitate apex, rarely fusiform or clavate, colorless or with brownish intracellular pigment, thin-walled. Pileipellis a trichohymeniderm, made up of cylindrical or narrowly clavate elements, 25–85 × 8–15 µm, with brown intracellular pigment, slightly thick-walled. Stipitipellis a cutis of cylindrical, thick-walled and incrusted, 5.5–10 µm wide hyphae. Caulocystidia numerous, 25–63 × 8–17 µm, in bundles, mostly clavate or cylindrical, sometimes utriform, with brown intracellular pigment, slightly thick-walled. Clamp connections absent in all tissues.

Habitat: solitary or in small group, on litter and rotten wood.

Collections examined: Vietnam, Central Highlands, Dalat Plateau, Lam Dong Province, Lac Duong District, Bidoup—Nui Ba National Park, 5 km of northeast Long Lanh Village, 12°10′10.837″ N, 108°41′55.183″ E, h = 1500 m, riparian tropical lower mountain evergreen forest dominated by species from the families *Fagaceae*, *Lauraceae*, *Magnoliaceae*, *Theaceae*, and *Podocarpaceae*, on litter, 13 November 2012, A.V. Alexandrova (LE 313055); Dak Nong Province, Dak Glong District, Ta Dung National Park, south-eastern slope of the Ta Dung Mt, 11°51′27.648″ N, 108°03′51.480″ E, h = 1415 m.a.s.l., evergreen broad-leaved forest (with *Lithocarpus* spp., *Quercus* sp., *Schima* sp., *Dilleniaceae*, *Myristicaceae*, and *Acer flabellatum*), on rotten wood, 15 October 2022, col. V.A. Dudka (LE F-347428).

Notes: *Pluteus* cf. *velutinus* was recently found in Vietnam [2], and we have now updated the description of the Vietnamese material based on additional collection from a neighboring province in the southern part of the country.

Compared to the previously studied specimen, the new collection differs in the shape of the pleurocystidia, which are predominantly broadly utriform, broadly clavate or spatuliform, usually without apical protrusions, however, other microscopic characters are generally the same. Here, we give an illustration of the new collection (Figure 19), but provide a combined description based on two studied specimens.

The nrITS sequences of both Vietnamese specimens are almost identical to each other, and on the resulting phylogenetic tree (Figure 3) they form a monophyletic clade, sister to the *P. velutinus* clade, which includes the holotype.

A more detailed discussion and justification of the inconclusive taxonomic interpretation of the studied material is provided in a previously published article [2].

## 4. Discussion

According to our current molecular and morphological studies on the specimens collected from Vietnam, the species diversity of *Pluteus* in the region is underestimated and many more new species remain to be described. This tropical mountain region of Southeast Asia harbors a rich fungal diversity due to the diverse ecological niches, as revealed by recent studies focusing on other groups of larger fungi in this region, such as the boletoid genera and *Entoloma* [42,43,44,45,46]. This is probably also true for the genus *Pluteus*.

Previously, only four species of the genus *Pluteus* have been reported from Vietnam [10,11,12]. However, research in the last few years has increased this diversity to seventeen species [2]. This study makes it possible to add six more species to the total diversity of the genus.

In this paper, twelve species of *Pluteus* are detected based on molecular and morphological data. Four species (*P. conformis*, *P. lucidus*, *P. ornatus* and *P. subroseus*) are described as new to science. Several taxa (*P.* aff. *septocystidiatus*, *P.* aff. *pauperculus* and *P.* cf. *velutinus*) are recognized as separate lineages from known species. The phylogenetic data presented above and inferred from nrITS and *tef1* sequences generally support the recognition of species circumscribed by macro- and micromorphological characteristics, and in many cases can be used to delimit clades of taxa with shared morphological features.

Many of the studied species occur in montane tropical broad-leaved forests, mainly in the southern (Dak Nong, Dong Nai, Lam Dong) and northern (Lao Cai, Nghe An) provinces of the country, but there are also some collections found in the central region (Quang Nam Province). Most species described here inhabit decomposed wood (stumps, trunks, and branches) of angiosperms, sometimes sawdust or wooden chips; only *P. chrysaegis* was found on a living tree in a coffee plantation. Ecology and geographical distribution of all species considered require further study, as many species are known so far only from their type localities, or some species are known due to few scattered finds.

Interestingly, based on phylogenetically confirmed results, *P. chrysaegis* is widely distributed but rare in the United States, Equatorial and West Africa, India, China and Vietnam. We expected to find *P. septocystidiatus*, which was originally described from Korea. At the same time, however, many *Pluteus* collections, emphasizing the high uniqueness of the Vietnamese mycobiota, leave many questions about the origin of species and the biogeographic relationships of the region. Thus, two collections (*P.* aff. *velutinus* and *P.* aff. *septocystidiatus*) are phylogenetically distinct from the known species, while the findings of *Pluteus* sp. 1 and *P.* aff. *pauperculus* suggest a common origin of some taxa from Vietnam, Australia, and Melanesia. Hence, the historical migration pathways remain to be studied.

Therefore, to assess the real species diversity of the genus *Pluteus*, it is necessary to continue intensive research in this biodiversity-rich region.

## Data Availability

Publicly available datasets were analyzed in this study. Those data can be found here: https://www.ncbi.nlm.nih.gov, accessed on 1 April 2023; https://www.mycobank.org, accessed on 1 April 2023.

## Supplementary Materials

The following supporting information can be downloaded at: https://www.mdpi.com/article/10.3390/jof9050584/s1, Table S1. List of species used in the phylogenetic analyses.
