# Supplementary material for: Observations on Pluteaceae in Vietnam: Four New Species and New Records of Pluteus
Source: J Fungi (Basel). 2023 May 18;9(5):584. doi: 10.3390/jof9050584 (PMC10221287; doi:10.3390/jof9050584)
Supplement: Supplementary file 1 [file jof-09-00584-s001.zip › jof-2365781-supplementary.pdf]

Journal of Fungi

MDPI

Table S1. List of species used in the phylogenetic analyses.

Species	Voucher/strain	Geographic origin	GenBank accession number	
			ITS	TEF1
Agaricus petasatus	LE 289372 Neotype	Russia	KJ009705	KJ009952
Pluteus albidus	SFSU:BAP 612	San Tome and Principe	MG968798	-
Pluteus albostipitatus	FK1891	Brazil	JQ801373	KJ010043
Pluteus albostipitatus	FK1799	Brazil	JQ801375	-
Pluteus alniphilus	PC0086107 Holotype	France	KJ009678	-
Pluteus americanus	AHS57842 Holotype	USA	KJ009762	KJ010037
Pluteus americanus	LE 289369	Russia	KJ009759	KJ010034
Pluteus atromarginatus	LE 289425	Russia	KJ009770	KJ010012
Pluteus aureovenatus	SP393697 Holotype	Brazil	FJ816663	-
Pluteus austrofulvus	AJ857 Holotype	USA	KM983701	-
Pluteus austrofulvus	iNaturalist 112280046	USA	ON864097	-
Pluteus austrofulvus	iNaturalist 112219822	USA	ON864096	-
Pluteus brunneidiscus	LE 289397	Russia	KJ009693	KJ009832
Pluteus brunneidiscus	AJ586	USA	KJ009694	KJ009834
Pluteus cervinus	REG 13641 Epitype	Germany	HM562152	KJ009860
Pluteus cervinus	LE 289396	Russia	KJ009626	KJ009848
Pluteus cervinus	AJ590	USA	KJ009628	KJ009878
Pluteus chrysaegis	FLAS-F-60411	USA	MF153092	-
Pluteus chrysaegis	GDGM:42376	China	MH059514	-
Pluteus chrysaegis	K13587	India	JN603206	-
Pluteus chrysaegis	JAD 248 (SFSU)	Vanuatu	OM060365	-
Pluteus chrysaegis	JAD 217 (SFSU)	Vanuatu	OM060364	-
Pluteus chrysaegis	**LE F-347436**	**Vietnam**	**OQ732741**	**OQ772318**
Pluteus concentricus	PDD_72493	New Zealand	MN738615	-
Pluteus concentricus	PDD_72484	New Zealand	MN738613	-
Pluteus concentricus	PDD:87570	New Zealand	MN738631	-
Pluteus conformis	**LE F-313664 Holotype**	**Vietnam**	**OQ732733**	**OQ772323**
Pluteus conformis	**LE F-313665**	**Vietnam**	**OQ732734**	**OQ772324**
Pluteus conformis	**LE F-313663**	**Vietnam**	**OQ732735**	**OQ772325**
Pluteus conizatus var. *africanus*	Thoen 5250	Dem. Rep. Congo	HM562142	-
Pluteus conizatus	JAD 245 (SFSU)	Vanuatu	OM060369	-
Pluteus conizatus	JAD 244 (SFSU)	Vanuatu	OM060368	-
Pluteus decoloratus	PDD 110522	New Zealand	MN738677	-
Pluteus decoloratus	JAC14640	New Zealand	MN738654	-
Pluteus densifibrillosus	SP393696 Holotype	Brazil	HM562159	KJ010041
Pluteus dianae	PR 629413 Holotype	Czech Republic	MH656433	-
Pluteus dianae	LE 303485	Russia	MH656431	-

Pluteus dianae	LE 312950	Russia	MH656430	-
Pluteus elaphinus	AJ783 Holotype	USA	KJ009663	KJ009902
Pluteus elaphinus	MSP	Canada	KJ009782	-
Pluteus eos	AJ589 Holotype	USA	KJ009683	KJ009808
Pluteus eos	LE 289379	Russia	KJ009675	KJ009807
Pluteus exilis	N5105 Holotype	USA	KJ009778	-
Pluteus exilis	MSSF87	USA	KF306020	-
Pluteus fernandezianus	JAD 331 (SFSU)	Vanuatu	OM060370	-
Pluteus fibrillosus	FK1903	Brazil	KR022018	-
Pluteus fulvibadius	Mushroom Observer 270623	USA	ON864093	-
Pluteus fulvibadius	AJ815	USA	KM983698	-
Pluteus glaucotinctus	SP394384	Brazil	HM562157	-
Pluteus glaucotinctus	Thoen 5546	Dem. Rep. Congo	HM562132	-
Pluteus glaucotinctus	Gossens-Fontana 5274 Holotype	Dem. Rep. Congo	HM562131	-
Pluteus granularis	SF20	USA	HM562189	-
Pluteus granularis	Strack7	USA	HM562069	-
Pluteus granulatus	HS18 2	Czech Republic	MH656435	-
Pluteus granulatus	HS11	Czech Republic	MH656434	-
Pluteus granulatus	OKA-349	Turkey	MG544916	-
Pluteus granulatus	AJ203	Spain	HM562048	-
Pluteus granulatus	LE 212990	Russia	FJ774086	-
Pluteus griseodiscus	GDGM42280 Holotype	China	KR350490	-
Pluteus griseodiscus	GDGM42373	China	KR350492	-
Pluteus harrisii	SP393709	Brazil	FJ816666	-
Pluteus harrisii	SP393708	Brazil	FJ816654	-
Pluteus heteromarginatus	AJ172	USA	HM562058	-
Pluteus hibbettii	AJ794 Holotype	USA	KJ009685	KJ009814
Pluteus hongoi	Singer A4070 Holotype	Japan	KJ009564	KJ009940
Pluteus hongoi	TENN 48297	China	KJ009613	KJ009932
Pluteus hongoi	S.D. Russell iNaturalist # 13060431	USA	ON006948	-
Pluteus hongoi	LE 289420	Russia	KJ009571	KJ009945
Pluteus hongoi	LE 289413	Russia	KJ009609	KJ009939
Pluteus hongoi	LE 234781	Russia	KJ009569	KJ009930
Pluteus hongoi	LE 9746	USA	KJ009574	KJ009904
Pluteus leucoborealis	LE 289421 Holotype	Russia	KJ009746	KJ009994
Pluteus leucoborealis	LE 289424	Mongolia	KJ009743	KJ009999
Pluteus leucoborealis	LE 289373	Russia	KJ009734	KJ009992
Pluteus longistriatus	LE 312951	Russia	KX216355	-
Pluteus longistriatus	FLAS-F-61188	USA	MH211798	-

Pluteus longistriatus	FG11092016010	Italy	MK446326	-
Pluteus longistriatus	KA17-0331	South Korea	MN294886	-
Pluteus longistriatus	ASIS24529	South Korea	KM052568	-
Pluteus longistriatus	SP394004	Brazil	HM562149	-
Pluteus longistriatus	SP394386	Brazil	HM562172	-
Pluteus losulus	GDGM47122	China	MH231231	-
Pluteus losulus	SFSU:DED 8313	San Tome and Principe	MG968803	-
Pluteus lucidus	**LE F-347426 Holotype**	**Vietnam**	**OQ732746**	**OQ772316**
Pluteus methvenii	A.S. Methven PNC1	USA	KJ00968	KJ009826
Pluteus methvenii	090926_AV04	Canada	KJ009793	KJ009829
Pluteus microspermus	JAC15434	New Zealand	MN738680	-
Pluteus microspermus	PDD_108760	New Zealand	MN738669	-
Pluteus minor	JAC14348	New Zealand	MN738650	-
Pluteus minor	JAC14793	New Zealand	MN738660	-
Pluteus minor	PDD 116894	New Zealand	MN738682	-
Pluteus neochrysaegis	JAD 265 (SFSU)	Vanuatu	OM060372	-
Pluteus olivaceofibrillosus	LE 313059	Vietnam	MT611230	-
Pluteus orestes	UC 1998591	USA	JX857470	KJ460256
Pluteus orestes	MO80923	USA	KJ009673	KJ009825
Pluteus oreibatus	ECV4183 Holotype	USA	KJ009763	KJ010033
Pluteus ornatus	**LE F-347437 Holotype**	**Vietnam**	**OQ732738**	**OQ772319**
Pluteus parvicarpus	LE 313357 Holotype	Russia	ON864114	-
Pluteus parvicarpus	LE 313631	Russia	ON864115	-
Pluteus parvisporus	iNaturalist 112236342 Holotype	USA	ON864098	-
Pluteus parvisporus	AJ 855	USA	ON864099	-
Pluteus pauperculus	JAC11068	New Zealand	MN738636	-
Pluteus pauperculus	JAC9790	New Zealand	MN738621	-
Pluteus* aff. *pauperculus	**LE F-347433**	**Vietnam**	**OQ732747**	**OQ772313**
Pluteus* aff. *pauperculus	**LE F-347434**	**Vietnam**	**OQ732748**	**OQ772314**
Pluteus pellitus	AJ72	Italy	HM562036	KJ009988
Pluteus pellitus	LE 289374	Russia	KJ009698	KJ009984
Pluteus pellitus	AJ74	Spain	HM562047	KJ009985
Pluteus petasatus	LE 289423	Mongolia	KJ009721	KJ009953
Pluteus petasatus	LOU: 7570	Spain	KJ009701	KJ009964
Pluteus plautus	UBCF23774	Canada	KC581304	-
Pluteus cf. *plautus*	UBCF23908	Canada	KJ146724	-
Pluteus podospileus	TNSF12398	Japan	HM562122	-
Pluteus podospileus	AJ782	USA	KM983687	-
Pluteus podospileus	AJ617	USA	KM983686	-
Pluteus podospileus	PDD: 86876	New Zealand	MN738618	-
Pluteus podospileus	AJ204	Spain	HM562049	-

Pluteus podospileus	LE 303682	Russia	KX216331	-
Pluteus podospileus	LE 303687	Russia	KX216332	-
Pluteus aff. *podospileus*	GM152	UK	KM983690	-
Pluteus aff. *podospileus*	MO136488	USA	KM983689	-
Pluteus podospilloides	LE 313230 Holotype	Vietnam	MT611236	-
Pluteus podospilloides	**LE F-313666**	**Vietnam**	**OQ732745**	**-**
Pluteus podospilloides	**LE F-347430**	**Vietnam**	**OQ732744**	**OQ772315**
Pluteus podospilloides	**LE F-347431**	**Vietnam**	**OQ732743**	**-**
Pluteus pouzarianus	LE 289380	Russia	KJ009668	…
Pluteus pouzarianus	PRM: 898519	Czech Republic	KJ009670	KJ009800
Pluteus pouzarianus	REG 13626	Germany	HM562154	KJ009806
Pluteus primus	A. Voitk 04-08-17-AV02	Canada	KJ009780	KJ009824
Pluteus primus	REG 13620	Germany	HM562167	KJ009821
Pluteus primus	LE 289390	Russia	KJ009680	KJ009818
Pluteus pulcherrimus	MCVE30061 Holotype	Italy	MK446327	-
Pluteus punctatus	PRM 682743 Holotype	Czech Republic	MH656438	-
Pluteus punctatus	JHC04 298	Sweden	MH656480	-
Pluteus purpureofuscus	HKAS48956 Holotype	China	KR350489	-
Pluteus rangifer	LE 203210 Holotype	Russia	KJ009650	KJ009898
Pluteus rangifer	LE 289385	Russia	KJ009651	KJ009896
Pluteus readiarum	PDD 103772	New Zealand	MN738644	-
Pluteus readiarum	JAC11228	New Zealand	MN738638	-
Pluteus romellii	BRNM 761731 Epitype	Czech Republic	ON864065	-
Pluteus romellii	BRNM 825846	Czech Republic	ON864067	-
Pluteus romellii	BRNM 825845	Slovakia	ON864070	-
Pluteus romellii	TNSF12387	Japan	HM562123	-
Pluteus salicinus	LE 202301	Russia	KJ009755	KJ010023
Pluteus salicinus	MA67874	Spain	HM562051	KJ010029
Pluteus salicinus	LE 213023	Russia	KJ009757	KJ010027
Pluteus semibulbosus	OKA-430	Turkey	MG544920	-
Pluteus semibulbosus	OKA-TR06	Turkey	MK123344	-
Pluteus semibulbosus	LE 312914	Russia	KX216353	-
Pluteus semibulbosus	A5058211	Spain	KR022020	-
Pluteus semibulbosus	SFC20150903-15	Korea	MF437007	-
Pluteus semibulbosus	ASIS22533	Korea	KF668315	-
Pluteus semibulbosus	**LE F-313653**	**Vietnam**	**OQ732740**	**-**
Pluteus aff. *semibulbosus*	TNSF12393	Japan	HM562090	-
Pluteus aff. *semibulbosus*	JAD 197 (SFSU)	Vanuatu	OM060359	-
Pluteus sepiicolor	LE 289365 Holotype	Russia	KJ009765	KJ010030
Pluteus sepiicolor	LE 289366	Russia	KJ009766	KJ010031
Pluteus septocystidiatus	AJ154	USA	HM562057	KJ010039
Pluteus septocystidiatus	AJ15	USA	HM562057	KJ010039

Pluteus septocystidiatus	**LE F-313668**	**Vietnam**	**OQ732736**	**OQ772321**
Pluteus septocystidiatus	BRNM_761662 Holotype	South Korea	HG964401	-
Pluteus septocystidiatus	AJ187	USA	HM562106	KJ010040
Pluteus* aff. *septocystidiatus	**LE F-313667**	**Vietnam**	**OQ732737**	**OQ772322**
Pluteus seticeps	Shaffer798	USA	HM562199	-
Pluteus seticeps	SF23	USA	HM562191	-
Pluteus seticeps	iNaturalist 129765552	USA	OP643122	-
Pluteus seticeps	iNaturalist 15114066	USA	OP541808	-
Pluteus shikae	TNSF12349 Holotype	Japan	HM562093	KJ009837
Pluteus shikae	LE 289383	Russia	KJ009696	KJ009835
Pluteus siccus	LE 313356 Holotype	Russia	ON864113	-
Pluteus subcervinus	LE 313525	Vietnam	MT611238	-
Pluteus subcervinus	K90736	India	KJ009752	KJ010010
Pluteus sublaevigatus	SP393694	Brazil	FJ816667	-
Pluteus subroseus	**LE F-347429 Holotype**	**Vietnam**	**OQ732739**	**OQ772317**
Pluteus umbrosoides	LE 312920	Russia	KX216349	-
Pluteus umbrosoides	LE 312839	Russia	KX216348	-
Pluteus umbrosoides	GDGM:29469	China	MH059512	-
Pluteus umbrosus	12187	Italy	JF908622	-
Pluteus umbrosus	LE 303696	Russia	KX216320	-
Pluteus umbrosus	MCVE30070	Italy	MK446324	-
Pluteus variabilicolor	OKA 116	Turkey	MG544908	-
Pluteus variabilicolor	LE 216873	Russia	FJ774077	-
Pluteus variabilicolor	TNSF17081	Japan	HM562099	-
Pluteus vellingae	AFTOL-ID 625 Holotype	USA	AY854065	-
Pluteus vellingae	BRNM 817769	Czech Republic	ON864108	-
Pluteus velutinus	MCVE29376	Italy	MG574947	-
Pluteus velutinus	TNSF12365	Japan	HM562114	-
Pluteus velutinus	LE 312913	Russia	KX216351	-
Pluteus velutinus	LE 289495	Mongolia	KX216341	-
Pluteus velutinus	MG574947	Italy	MK446331	-
Pluteus velutinus	LE 303693	Russia	KX216340	-
Pluteus velutinus	FK1889	Brazil	KR022027	-
Pluteus velutinus	TNSF12372	Japan	HM562127	-
Pluteus velutinus	K12851 Holotype	India	JN603205	-
Pluteus velutinus	MCVE29375	Italy	MG576118	-
Pluteus velutinus	LE 312915	Russia	KX216352	-
Pluteus cf. *velutinus*	LE 313055	Vietnam	MT611241	-
Pluteus* cf. *velutinus	**LE F-347428**	**Vietnam**	**OQ732742**	**-**
Pluteus xylophilus	SP393707	Brazil	HM562163	-
Pluteus xylophilus	NMJ147	Brazil	FJ816660	-
***Pluteus* sp. 1**	**LE F-313670**	**Vietnam**	**OQ732732**	**OQ772320**

Pluteus sp.	66A	USA	JQ775567	-
Pluteus sp.	JAD 189 (SFSU) Holotype	Vanuatu	OM060387	-
Pluteus sp.	JAD 169 (SFSU)	Vanuatu	OM060389	-
Pluteus sp.	JAD 229 (SFSU)	Vanuatu	OM060391	-
Pluteus sp.	JAD 160 (SFSU)	Vanuatu	OM060388	-
Pluteus sp.	GDGM 42371	China	KU382739	-
Pluteus sp.	GDGM 41576	China	KU382738	-
Pluteus sp.	AJ606	USA	KR022011	-
Pluteus sp.	UC 1861124	USA	KC147672	-
Pluteus sp.	MO93671	USA	KR022009	-
Pluteus sp.	PDD 107523	New Zealand	KU131669	-
Pluteus sp.	TENN055370	Argentina	KY352653	-
Pluteus sp.	FLAS-F-61719	USA	MH212067	-

Species names in bold are newly generated sequences for this study.
